# Synthesis and *In Vivo* Evaluation of a Site-specifically Labeled Radioimmunoconjugate for Dual-Modal (PET/NIRF) Imaging of MT1-MMP in Sarcomas

**DOI:** 10.1021/acs.bioconjchem.2c00306

**Published:** 2022-07-22

**Authors:** Toni A. Pringle, Corey D. Chan, Saimir Luli, Helen J. Blair, Kenneth S. Rankin, James C. Knight

**Affiliations:** † School of Natural and Environmental Sciences, Newcastle University, Newcastle Upon Tyne NE1 7RU, U.K.; ‡ Newcastle Centre for Cancer, Newcastle University, Newcastle Upon Tyne NE1 7RU, U.K.; § North of England Bone and Soft Tissue Tumour Service, Newcastle Upon Tyne Hospitals NHS Foundation Trust, Freeman Road, Newcastle Upon Tyne NE7 7DN, U.K.; ∥ Translational and Clinical Research Institute, Newcastle University, Newcastle Upon Tyne NE1 7RU, U.K.; ⊥ Preclinical In Vivo Imaging, Translational and Clinical Research Institute, Newcastle University, Newcastle Upon Tyne NE2 4HH, U.K.; # Wolfson Childhood Cancer Research Centre, Newcastle Upon Tyne NE1 7RY, U.K.

## Abstract

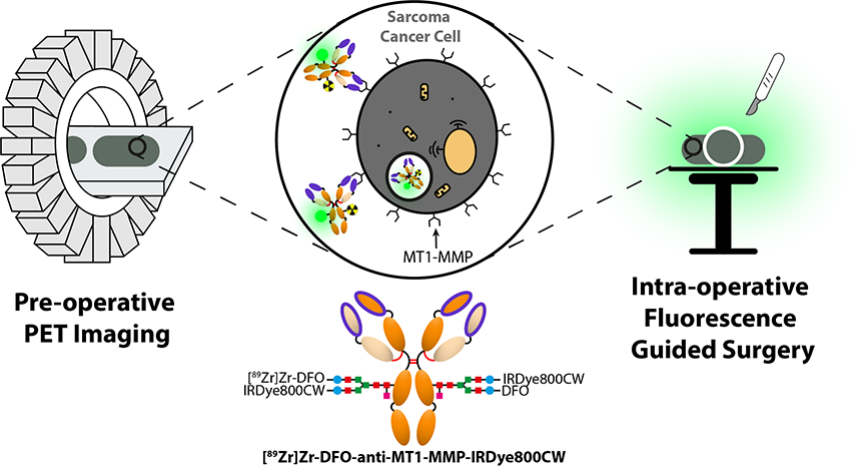

Bone sarcomas are devastating primary bone cancers that mostly affect children, young adults, and the elderly. These aggressive tumors are associated with poor survival, and surgery remains the mainstay of treatment. Surgical planning is increasingly informed by positron emission tomography (PET), and tumor margin identification during surgery is aided by near-infrared fluorescence (NIRF) imaging, yet these investigations are confounded by probes that lack specificity for sarcoma biomarkers. We report the development of a dual-modal (PET/NIRF) immunoconjugate ([^89^Zr]Zr-DFO-anti-MT1-MMP-IRDye800CW) that targets MT1-MMP, a matrix metalloproteinase overexpressed in high-grade sarcomas. [^89^Zr]Zr-DFO-anti-MT1-MMP-IRDye800CW was synthesized *via* site-specific chemoenzymatic glycan modification, characterized, and isolated in high specific activity and radiochemical purity. Saturation binding and immunoreactivity assays indicated only minor perturbation of binding properties. A novel mouse model of dedifferentiated chondrosarcoma based on intrafemoral inoculation of HT1080 WT or KO cells (high and low MT1-MMP expression, respectively) was used to evaluate target binding and biodistribution. Fluorescence and Cerenkov luminescence images of [^89^Zr]Zr-DFO-anti-MT1-MMP-IRDye800CW showed preferential uptake in HT1080 WT tumors. *Ex vivo* gamma counting revealed that uptake in MT1-MMP-positive tumors was significantly higher than that in control groups. Taken together, [^89^Zr]Zr-DFO-anti-MT1-MMP-IRDye800CW is a promising dual-modal sarcoma imaging agent for pre-operative surgical planning and intraoperative surgical guidance.

## Introduction

Bone sarcomas are a group of rare, heterogeneous cancers associated with poor clinical outcomes that predominantly affect children and young adults, as well as the elderly.^1^ The mainstay of treatment for bone sarcomas is surgical resection, alongside chemotherapy and radiotherapy depending on the tumor type; however, despite imaging and treatment advancements, the 5 year overall survival rate has remained similar for the past 30 years (53–55%).^2^ In older patients, dedifferentiated chondrosarcoma is a particularly aggressive form of bone sarcoma with a 5 year survival rate of just 18%.^3^

Patients with primary malignant bone tumors often present with persistent pain, leading to X-ray and, preferably, MRI investigations.^2^ Surgery with or without adjuvant therapy is the mainstay of treatment for localized or resectable bone sarcomas.^2,5^ Pre-operative imaging is performed to locate the tumor and optimize margins for excision. This is currently often accomplished using two plane radiographs, along with MRI and CT scans in most cases. Positron emission tomography (PET) scanning with the glucose metabolism radiotracer ^18^F-fluorodeoxyglucose ([^18^F]FDG) has been evaluated in pre-operative imaging of sarcomas in a recent clinical study.^6^ The results from pre-operative imaging are discussed at multidisciplinary team meetings to determine the best surgical procedure for each patient, given the high variability between cases.^2^ Surgical resection aims to remove the tumor fully surrounded by healthy tissue to achieve negative tumor margins, while maintaining as much healthy tissue as possible to maximize the recovery and functional outcomes for the patient. Following tumor resection, histological assessment is performed to ensure that negative margins have been achieved. A negative margin means that the tumor has been removed completely with no cancer left behind. If the margin is positive, this indicates that the patient may need further surgery to remove any residual cancer cells. It is important to achieve negative margins at the first operation as the risk of bone sarcoma recurrence has been found to strongly correlate to the incidence of positive tumor margins after excision.^7^ Although pre-operative imaging techniques can provide good tumor margin identification, it can be difficult to refer to these images during open resection due to the changing anatomy intraoperatively.^8^

Intraoperative fluorescence-guided surgery (FGS) can aid surgeons by providing real-time guidance in discerning tumor margins using near-infrared cameras. Indocyanine green (ICG) is a well-established near-infrared fluorescence (NIRF) dye that has recently been used for intraoperative image-guided sarcoma resection^9^ and has shown potential to reduce the unexpected positive margin rate.^10^ The preferential tumor uptake of ICG is non-targeted, occurring due to the enhanced permeability and retention effect.^11^ Due to its lack of specificity, ICG is limited by issues surrounding off-target and background fluorescence as the dye can accumulate in necrotic, non-cancerous, and inflamed tissues.^8,12^ Alternatively, targeted approaches can be used to overcome these issues by conjugating the fluorophore to a suitable biomarker-specific vector, such as an antibody.^13^ However, the lack of clinically validated sarcoma-specific biomarkers has hampered progress in this area.

Membrane type-1 matrix metalloproteinase (MT1-MMP, also known as MMP-14) is a cell surface enzyme overexpressed in a range of cancers, including sarcoma, and is associated with tumor growth, invasion, metastasis, and angiogenesis.^14^ MT1-MMP belongs to a family of MMPs that degrade components of the extracellular matrix (ECM).^15^ The principal role of MT1-MMP involves degradation and remodeling of collagen and proteoglycans; however, it also activates other proteolytic MMPs, which themselves degrade ECM components, leading to increased invasion of the basement membrane. In addition, MT1-MMP induces epithelial–mesenchymal transition, a process by which cancer cells undergo changes in their morphology, leading to a migratory phenotype,^16^ promoting extravasation into adjacent blood vessels and metastasis to distant tissues.^17^ Lastly, MT1-MMP binding to the tissue inhibitor of metalloproteinases 2 (TIMP-2) increases expression of MMP-2, which is associated with a poor response to chemotherapy in some bone sarcoma subtypes.^18^ MT1-MMP therefore represents an attractive imaging biomarker of sarcomas.

A murine anti-MT1-MMP monoclonal antibody (LEM2/15) that binds to residues 218–233 of the V–B loop of MT1-MMP with high affinity (*K*_d_ = 0.4 nM) has been used in two recent studies to facilitate preclinical immunoPET imaging of MT1-MMP overexpression in pancreatic ductal adenocarcinoma and glioma.^19,20^ In both cases, the method for labeling LEM2/15 with the PET radiometal zirconium-89 (^89^Zr) was based upon conventional non-site-specific conjugation of an isothiocyanate-functionalized derivative of desferrioxamine (DFO) to the ε-amino group of any accessible lysine residue on the antibody, resulting in heterogeneous mixtures of products that can be difficult to chemically define due to highly variable degrees of labeling. Furthermore, this non-site-specific approach can lead to reductions in the immunoreactivity of the radioimmunoconjugate (RIC) due to modifications at the antigen binding domain. An alternative bioconjugation strategy based on site-specific chemoenzymatic modification of IgG heavy chain glycans was first applied to RICs by Zeglis *et al.*(21) This approach enables precise control of the conjugation sites and stoichiometry, resulting in a greatly reduced distribution of products, which is especially important in dual-modal RICs due to the attachment of two different reporter moieties. The administration of a single dual-modal antibody, in contrast to a mixture of monomodal radio- and fluorophore-labeled antibodies, is advantageous as it avoids differing pharmacokinetic profiles and ensures co-localization of signal from each modality. In addition, the synthesis, preclinical evaluation, and clinical translation of a single dual-modal antibody is more economical and less laborious relative to the development of two distinct agents.^22^

We report the development of a dual-modal (PET/NIRF) imaging agent based on a site-specifically glycoengineered anti-MT1-MMP (LEM2/15.8) immunoconjugate for pre-operative PET-based assessment and FGS of MT1-MMP-overexpressing sarcomas. Preclinical evaluation of [^89^Zr]Zr-DFO-anti-MT1-MMP-IRDye800CW was facilitated by radioligand binding assays, radio-immunohistochemistry, and *in vivo* experiments. In order to maximize the clinical translatability of our *in vivo* study, we developed a murine model of MT1-MMP-expressing dedifferentiated chondrosarcoma by intrafemoral orthotopic injection, allowing us to investigate the dual-modal agent with clinical relevance to the sarcoma field.

## Results and Discussion

### Synthesis and Characterization of Antibody Conjugates

**Figure 1:**
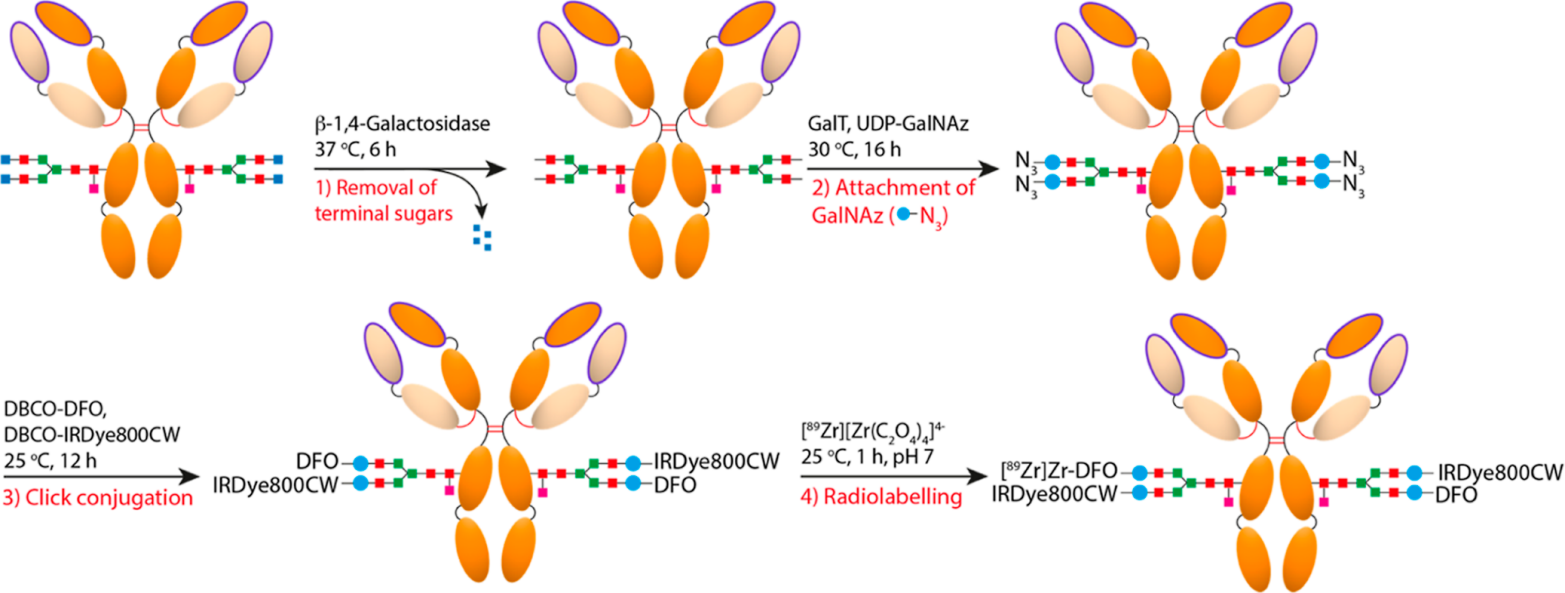
Scheme of the chemoenzymatic approach used to synthesize the dual-modal (PET/NIRF) RICs based on site-specific modification of the N-linked biantennary glycans situated on the heavy chain of the antibody.

Dual-labeled RICs were prepared by a four-step synthesis involving the chemoenzymatic modification of the heavy chain glycans of either anti-MT1-MMP or mouse IgG_1_ control antibodies with dibenzocyclooctyne (DBCO)-modified derivatives of DFO and IRDye800CW (Figure 1).

The overall synthetic yields of the radiolabeling precursors DFO-anti-MT1-MMP-IRDye800CW and DFO-IgG-IRDye800CW were 46.15 ± 10.14% (*n* = 7) and 68.44 ± 3.60% (*n* = 3), respectively. The degree of labeling of IRDye800CW (DOL_IRDye800CW_) for anti-MT1-MMP and IgG antibodies was determined using UV–Vis spectroscopy to be 1.74 ± 0.47 (*n* = 7) and 1.41 ± 0.68 (*n* = 3), respectively. High-performance liquid chromatography coupled with electrospray ionization-quadrupole-time of flight-mass spectrometry (LC ESI-QTOF MS) analysis of the conjugates was performed to assess the site-specific nature of the conjugation method (Figure S1). As expected, the spectra showed an increase in mass of the heavy chain of the azide and DFO/IRDye800CW-modified antibodies compared to the unmodified Ab. The light chain of all anti-MT1-MMP Ab samples remained the same mass throughout (24,080 Da).

### Radiolabeling and Characterization

Radiochemical yields (RCY) were determined by radio-instant thin layer chromatography (radio-iTLC) to be 66 ± 28% (*n* = 5) for the anti-MT1-MMP RIC and 74 ± 22% (*n* = 3) for the IgG control (Figure S2). Size exclusion chromatography enabled the isolation of both RICs in excellent radiochemical purity (>99%) and high specific activity (0.1 MBq/μg).^23^

**Figure 2:**
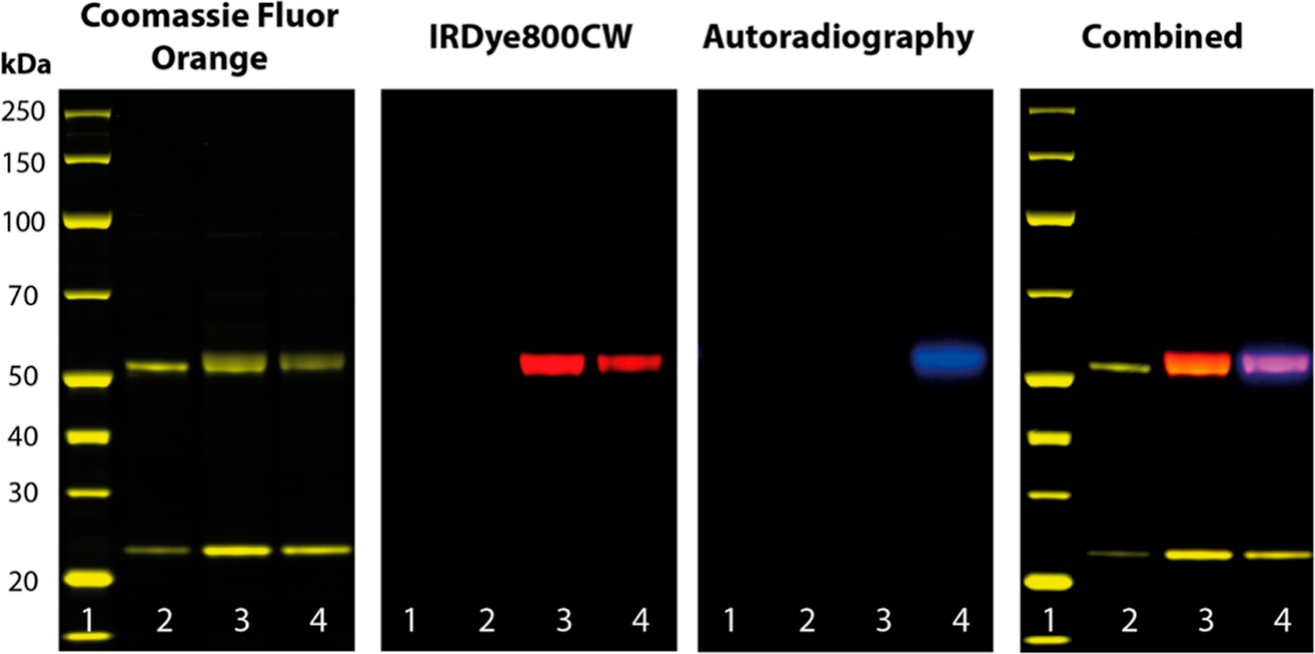
SDS-PAGE of unmodified anti-MT1-MMP (lane 2), DFO-anti-MT1-MMP-IRDye800CW (lane 3), and [^89^Zr]Zr-DFO-anti-MT1-MMP-IRDye800CW (lane 4) compared to a protein ladder (lane 1, masses shown on the left). The gel was run under reducing conditions, stained with Coomassie Fluor Orange, and analyzed by fluorescence imaging and autoradiography.

Site-specific modification of the N-linked glycans on the heavy chains of the anti-MT1-MMP antibody was confirmed by sodium dodecyl-sulfate polyacrylamide gel electrophoresis (SDS-PAGE) under reducing conditions (Figure 2). Fluorescence imaging and autoradiography analysis revealed co-localization of both IRDye800CW and ^89^Zr with the bands corresponding to the heavy chains (∼50 kDa), with no evidence of non-specific conjugation on the light chains (∼25 kDa).

The solution stability of [^89^Zr]Zr-DFO-anti-MT1-MMP-IRDye800CW in phosphate buffered saline (PBS), mouse serum, and human serum was assessed daily by radio-iTLC over 7 days at 25 and 37 °C. At 25 °C, the RIC remained >95% intact in all solutions over 7 days (Figure S3). At 37 °C, the stability of the RIC remained high (>80%) in both human and mouse serum over 7 days, although a decline in stability was evident in PBS from day 5 and only 49% of the RIC remained intact at day 7. This is most likely due to the absence of serum proteins in PBS, such as the free radical scavenger albumin, which would otherwise impart a minor radioprotective effect.^24^

**Figure 3:**
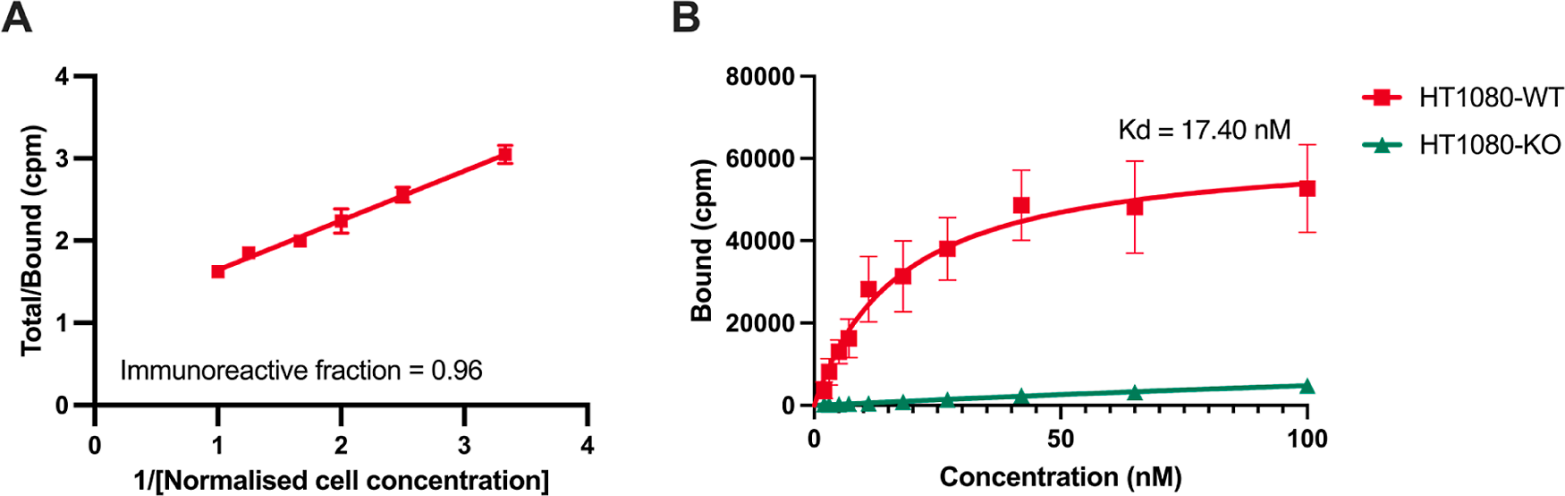
(A) Immunoreactivity of [^89^Zr]Zr-DFO-anti-MT1-MMP-IRDye800CW in HT1080 WT cells; (B) saturation binding plot to determine binding affinity (*K*_d_) of [^89^Zr]Zr-DFO-anti-MT1-MMP-IRDye800CW in HT1080 WT cells.

The immunoreactive fraction of [^89^Zr]Zr-DFO-anti-MT1-MMP-IRDye800CW was >95%, indicating that the site-specific bioconjugation strategy effectively preserved the antigen binding fragment with only minimal impact upon epitope binding (Figure 3A). Furthermore, the high binding affinity of [^89^Zr]Zr-DFO-anti-MT1-MMP-IRDye800CW determined in HT1080 WT cells (wild-type cells, high MT1-MMP expression; *K*_d_ = 17.4 nM) and negligible binding to HT1080 KO cells (MT1-MMP knock-out cells with low MT1-MMP expression, Figures S7–S14) indicate favorable specificity (Figure 3B). The saturation binding assay revealed a B_max_ (cpm) value of 63,216 for HT1080 WT cells, while uptake on HT1080 KO control cells could not be saturated due to very low MT1-MMP expression.

### *Ex Vivo* MT1-MMP Staining

**Figure 4:**
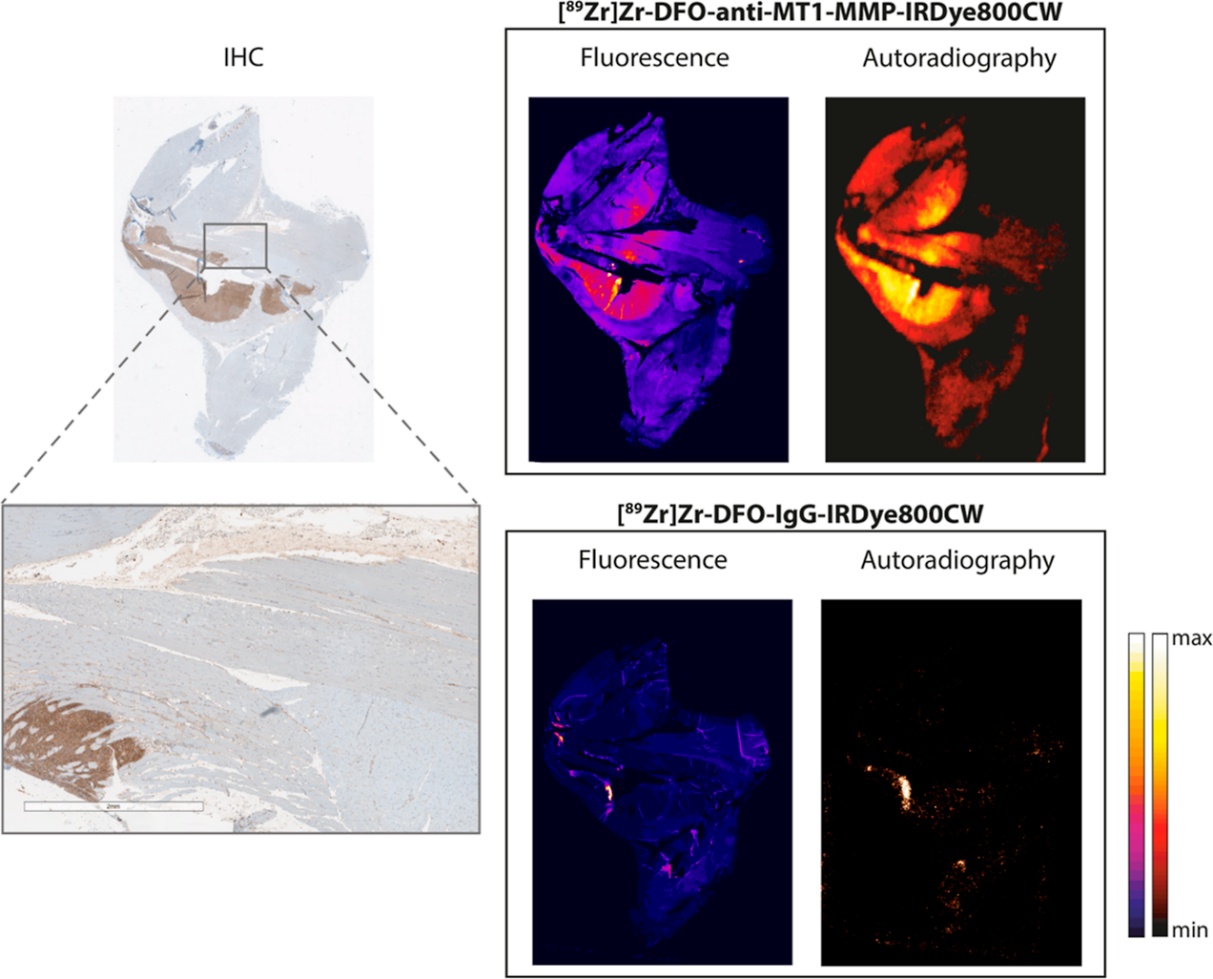
Representative immunohistochemistry (brown: MT1-MMP, blue: nucleus) with additional magnification, fluorescence, and digital autoradiography images of adjacent murine tumor sections stained with [^89^Zr]Zr-DFO-anti-MT1-MMP-IRDye800CW or [^89^Zr]Zr-DFO-IgG-IRDye800CW.

Immunohistochemistry, fluorescence, and digital autoradiography images of adjacent murine tumor sections incubated with [^89^Zr]Zr-DFO-anti-MT1-MMP-IRDye800CW were co-registered in order to assess the specificity of the dual-modal tracer for MT1-MMP (Figure 4). Excellent co-localization of both fluorescence (IRDye800CW) and radioactive (^89^Zr) signals can be observed with regions of elevated MT1-MMP expression on immunohistochemistry (IHC) images (Figure 4; brown), whereas regions without MT1-MMP show low, diffuse signal in both imaging modalities. Adjacent sections incubated with the non-specific control, [^89^Zr]Zr-DFO-IgG-IRDye800CW, showed no co-localization of fluorescence or radioactivity with MT1-MMP.

### *In Vivo* Evaluation

**Figure 5:**
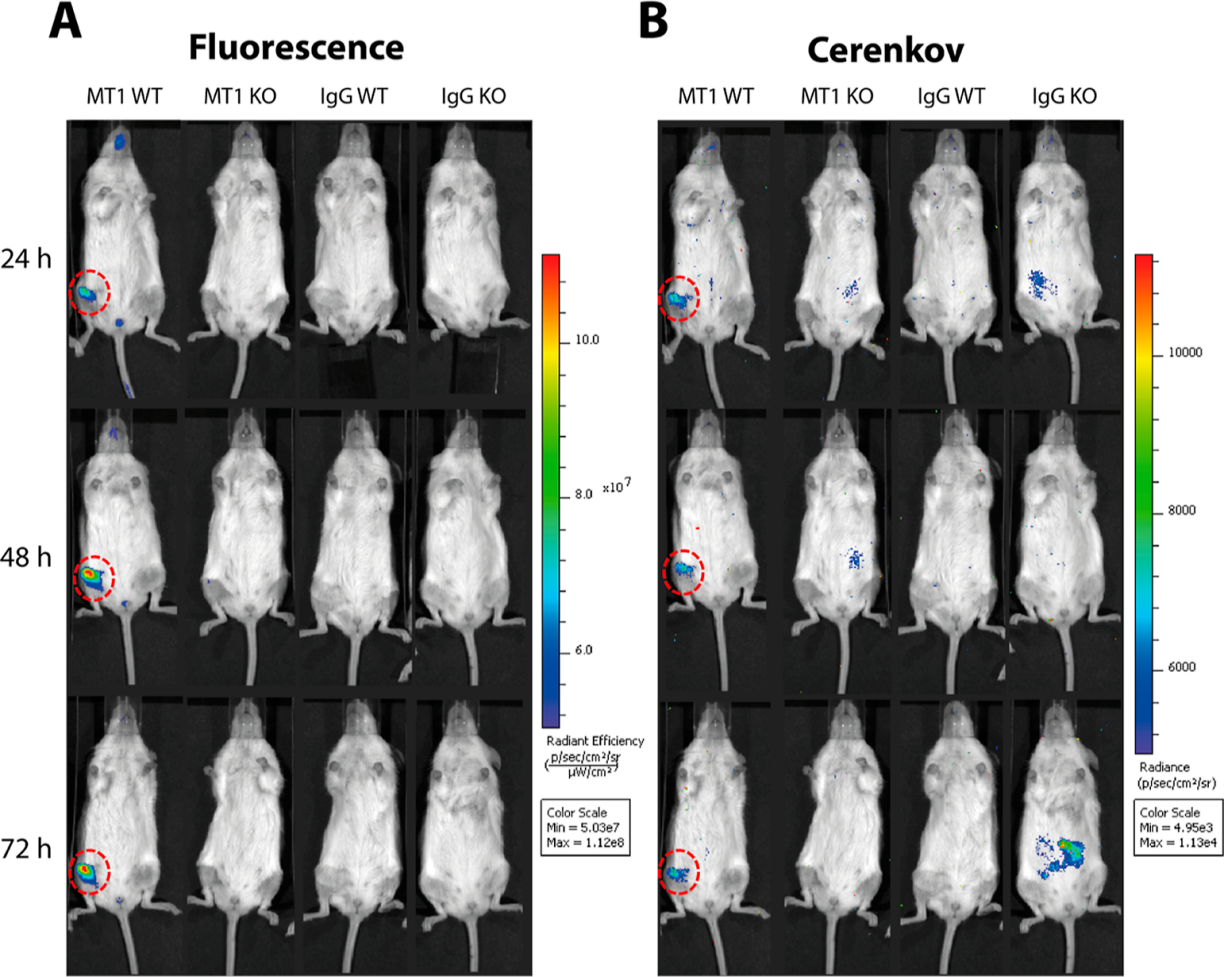
Representative IVIS images showing mice bearing either HT1080 WT or KO tumours (right femur in supine position; red dashed circle) acquired at 24, 48, and 72 h p.i. of either [^89^Zr]Zr-DFO-anti-MT1-MMP-IRDye800CW (MT1) or [^89^Zr]Zr-DFO-IgG-IRDye800CW (IgG). Mice were imaged for (A) fluorescence (*E*_x_ = 745 nm, *E*_m_ = 800 nm) and (B) Cerenkov luminescence (open filter).

To assess the ability of [^89^Zr]Zr-DFO-anti-MT1-MMP-IRDye800CW to visualize MT1-MMP expression *in vivo*, we developed a clinically relevant murine model of MT1-MMP positive and MT1-MMP negative dedifferentiated chondrosarcoma by orthotopic intrafemoral injection with either HT1080 WT or HT1080 KO cells, respectively. Using this model, intrafemoral tumors were scanned for fluorescence and radioisotope-generated Cerenkov luminescence at 24, 48, and 72 h following intravenous (IV) administration of [^89^Zr]Zr-DFO-anti-MT1-MMP-IRDye800CW or [^89^Zr]Zr-DFO-IgG-IRDye800CW. Cerenkov luminescence imaging was used as a rapid, simple, and cost-effective alternative to PET for semiquantitative *in vivo* evaluation of the RICs.^25^ HT1080 WT tumor-bearing mice that received the MT1-MMP-specific RIC showed higher fluorescence and Cerenkov signal at the tumor site compared to control groups at all time points (Figures 5A,B, S4). Moreover, images from both modalities showed a reduction in background signal at a rate that is typical of an intact IgG, leading to an enhancement of tumor contrast over 72 h.

**Figure 6:**
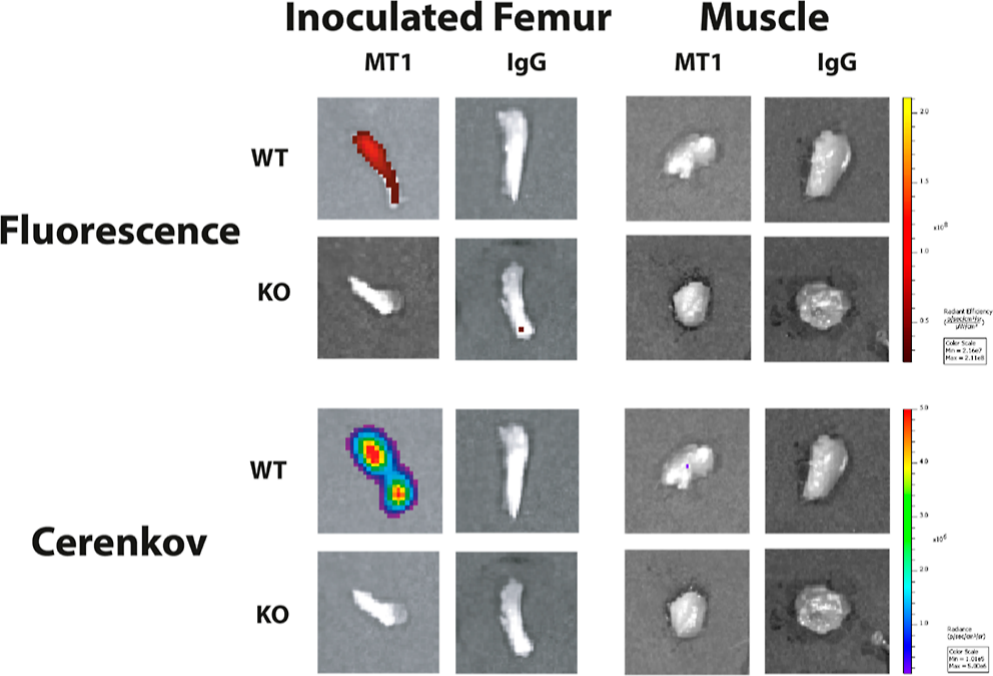
Representative fluorescence (top) and Cerenkov (bottom) images, respectively, showing the radiant efficiency and radiance of inoculated femurs (left) and contralateral thigh muscle (right) for all groups.

After the 72 h imaging timepoint, selected organs were excised and imaged for both Cerenkov luminescence and fluorescence (Figures 6, S5). Fluorescence intensity was significantly higher in HT1080 WT inoculated femurs of mice administered [^89^Zr]Zr-DFO-anti-MT1-MMP-IRDye800CW compared to controls (*P* < 0.05). Moreover, fluorescence signal was negligible in all other organs, which serves to highlight the potential utility of this RIC in FGS applications. Cerenkov images were consistent with these observations but were not quantified due to high tissue photon attenuation.^26^

**Figure 7:**
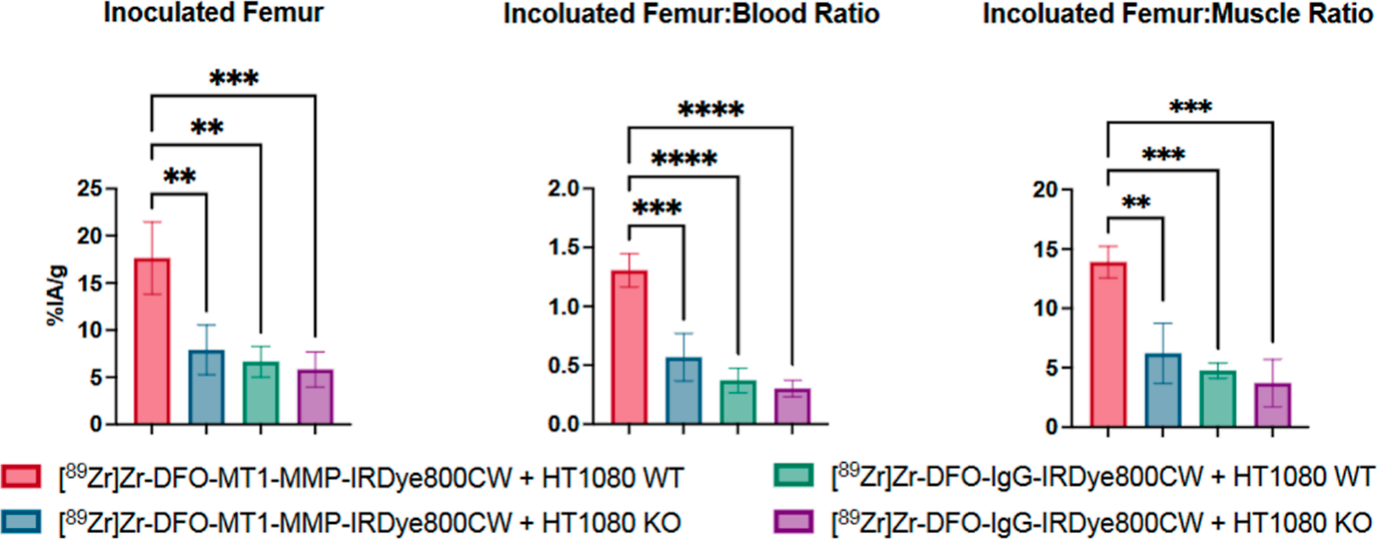
Biodistribution data collected 72 h after the administration of [^89^Zr]Zr-DFO-MT1-MMP-IRDye800CW or [^89^Zr]Zr-DFO-IgG-IRDye800CW in mice bearing HT1080 WT (high MT1-MMP) or HT1080 KO (low MT1-MMP) tumors. (A) Uptake (%IA/g) in the inoculated femur for all groups; (B) inoculated femur-to-blood ratios; (C) inoculated femur-to-muscle ratios. Each group contained at least 3 animals. *P < 0.05. **P < 0.01. ***P < 0.001. ****P < 0.0001. Error bars represent standard deviation.

The biodistribution of the RIC was assessed by gamma counting at 72 h p.i. (Table 1). Uptake of [^89^Zr]Zr-DFO-anti-MT1-MMP-IRDye800CW in HT1080 WT (high MT1-MMP) inoculated femurs (17.64 ± 3.84 %IA/g) was significantly higher compared to that in the HT1080 KO control group (7.09 ± 2.62 [*P* < 0.01]) and higher than uptake of the non-specific IgG control in both HT1080 WT and KO groups (6.65 ± 1.64 %IA/g [*P* < 0.01] and 5.82 ± 1.84 %IA/g [*P* < 0.001], respectively) (Figure 7; left). This uptake value is comparable to previously reported values obtained using the related agent ^89^Zr-DFO-LEM2/15 in a mouse model of glioma based on U251 xenografts (17.7 ± 2.6 and 14.3 ± 2.0 %IA/g at day 2 and 4 p.i., respectively)^20^ and higher than that observed in mice bearing CAPAN-2 xenografts (6.29 ± 0.64 %IA/g at day 3 p.i).^27^ Similarly, inoculated femur-to-blood (Figure 7; middle) and inoculated femur-to-muscle (Figure 7; right) uptake ratios for [^89^Zr]Zr-DFO-anti-MT1-MMP-IRDye800CW in HT1080 WT femurs (T/B: 1.31 ± 0.14; T/M: 13.89 ± 1.33) were significantly higher than that in control groups. Although the extent of muscle invasion varied in this model, increased uptake of [^89^Zr]Zr-DFO-anti-MT1-MMP-IRDye800CW was also observed in the thigh muscle immediately adjacent to femurs inoculated with HT1080 WT cells (Table 1, Figure S6). [^89^Zr]Zr-DFO-anti-MT1-MMP-IRDye800CW also accumulated highly in the spleen (27.90 ± 3.98 %IA/g), and overall, splenic uptake of the anti-MT1-MMP probe was significantly higher than that of the IgG (*P* < 0.001), suggesting that uptake is partly mediated by MT1-MMP. Indeed, MT1-MMP is highly expressed in spleen;^28^ however, it is also worth noting that high splenic uptake is often encountered in immunoPET studies involving immunodeficient mouse models due to antibody/Fc receptor interactions.^29^ No other statistically significant differences were apparent for any other organ, and the overall biodistribution profile is typical of an ^89^Zr-labeled antibody in mice at 3 days p.i.^30^

**Table 1:** *Ex vivo* Biodistribution Data (%IA/g ± S.D.) for the [^89^Zr]Zr-DFO- and IRDye800CW-Labeled anti-MT1-MMP and IgG RICs in NSG Mice Bearing Either HT1080 WT (High MT1-MMP) or HT1080 KO (Low MT1-MMP) Orthotopic Tumors

organ	[^89^Zr]Zr-DFO-MT1-MMP-IRDye800CW + HT1080 WT	[^89^Zr]Zr-DFO-MT1-MMP-IRDye800CW + HT1080 KO	[^89^Zr]Zr-DFO-IgG-IRDye800CW + HT1080 WT	[^89^Zr]Zr-DFO-IgG-IRDye800CW + HT1080 KO
blood	13.77 ± 4.30	14.24 ± 2.23	18.22 ± 2.13	19.11 ± 2.78
inoculated femur	17.64 ± 3.84	7.91 ± 2.62	6.65 ± 1.64	5.82 ± 1.85
invaded thigh muscle	19.28 ± 14.02	10.55 ± 11.83	7.21 ± 5.68	3.37 ± 2.96
contralateral femur	7.57 ± 2.79	6.00 ± 4.50	4.65 ± 0.89	4.11 ± 0.27
contralateral thigh muscle	1.28 ± 0.32	1.31 ± 0.14	1.38 ± 0.20	1.68 ± 0.34
lung	10.36 ± 1.16	8.29 ± 1.58	10.00 ± 3.06	8.17 ± 1.34
liver	12.89 ± 1.31	10.80 ± 1.43	9.51 ± 0.83	7.97 ± 1.34
heart	4.68 ± 1.44	5.46 ± 2.29	5.36 ± 0.49	4.68 ± 0.91
spleen	27.90 ± 3.98	26.29 ± 11.03	13.10 ± 3.37	9.30 ± 2.01
stomach	1.133 ± 0.33	0.68 ± 0.26	1.09 ± 0.38	0.98 ± 0.31
small intestine	1.99 ± 0.43	1.76 ± 0.19	1.83 ± 0.24	1.71 ± 0.42
large intestine	2.05 ± 0.38	1.90 ± 0.33	2.06 ± 0.64	1.74 ± 0.11
pancreas	3.11 ± 1.47	2.43 ± 0.95	2.97 ± 0.51	2.32 ± 0.34
kidneys	8.42 ± 1.62	7.67 ± 0.89	6.22 ± 0.68	5.24 ± 0.98
skin	3.78 ± 1.39	5.09 ± 2.21	3.28 ± 1.73	4.23 ± 2.81
fat	3.74 ± 1.48	4.80 ± 1.87	3.07 ± 2.92	3.30 ± 2.56

## Conclusions

This study provides data to support [^89^Zr]Zr-DFO-anti-MT1-MMP-IRDye800CW as a promising dual-modal imaging agent for pre-operative surgical planning and intraoperative FGS in sarcoma. Favorable MT1-MMP binding specificity has been demonstrated in both *in vitro* experiments and a novel *in vivo* mouse model of dedifferentiated chondrosarcoma. The development of anti-MT1-MMP RICs could overcome a major challenge in FGS of sarcoma that stems from the use of probes that lack specificity for molecular biomarkers of sarcoma, leading to improved resection margins and better patient outcomes. With a view to clinical translation, future work will evaluate the *in vivo* stability, toxicity, and dosimetry of the RIC and involve direct comparisons with other imaging agents including ICG and [^18^F]FDG. In addition, humanization of the anti-MT1-MMP antibody will be necessary to reduce the likelihood of adverse immunogenic reactions in patients. Lastly, the development of a good manufacturing practice compliant production method will be required for human administration and evaluation in early phase clinical trials to assess the feasibility, safety profile, and optimal dosing of [^89^Zr]Zr-DFO-anti-MT1-MMP-IRDye800CW for pre-operative PET imaging and FGS in patients with bone and soft tissue sarcomas.

## Experimental Procedures

### General Methods

All reagents were purchased from Thermo Fisher Scientific unless otherwise stated and used without further purification. Water was deionized using a Select Fusion ultrapure water deionization system (Suez) and had a resistance of >18.2 MΩ cm^–1^ at 25 °C. Protein concentration measurements were obtained using a NanoDrop One Microvolume UV–vis spectrophotometer (NanoDrop Technologies, Inc.). Mass spectrometry measurements were performed using a Thermo RSLC coupled to Bruker maXis. Radioactivity measurements were obtained using a CRC-25 Dose Calibrator (Capintec, Inc.) or a Wizard 2480 Gamma Counter (PerkinElmer). RIC synthesis and serum stability studies were monitored by instant thin-layer chromatography using glass microfiber chromatography paper (iTLC-SA, Agilent). Radio-iTLC strips were measured by autoradiography (Amersham Typhoon Bioimager, GE) and analyzed using ImageQuant software (GE Healthcare). All experiments were performed in accordance with the United Kingdom Human Tissue Act (2004) regulations. Appropriate informed consent for the use of human tumor specimen slides and paraffin embedded blocks was obtained and approved by the Newcastle and North Tyneside 1 Research Ethics Committee (REC reference number: 17/NE/0361).

### Site-Specific Antibody Modification

#### Preparation of the Azide-modified mAb

Azide-modified anti-MT1-MMP (MSX LEM-2/15.8, Millipore) or IgG (mouse IgG_1_ Isotype Control, Bio-techne, Catalog # MAB002) was prepared using the SiteClick Antibody Azido Modification Kit from Thermo Fisher using a slightly modified procedure. Briefly, a purified aliquot of anti-MT1-MMP or IgG (300 μg) was incubated with β-1,4-galactosidase (10 μL) at 37 °C for 6 h at 450 rpm in a reaction volume of 60 μL. A solution containing UDP-GalNAz (220 μg), GalT (Y289L) enzyme (80 μL), SiteClick buffer additive (25 μL), 20× tris buffer (12.5 μL, pH 7), and deionized water (75 μL) was then added to the antibody solution. The resulting mixture was incubated at 30 °C for 16 h at 450 rpm. The azide-modified antibody was then isolated from the mixture using pre-rinsed 30 kDa molecular weight cutoff 0.5 mL centrifugal filters (Amicon) at 12,000 × *g* for 10 min, followed by three washes and adjustment to 1 mg/mL using 50 mM Tris buffer (pH 7.0). To the azide-modified antibody solution, the following were added: (i) 15 M equivalents of DFO-DBCO (Macrocyclics) from a 2 mM stock solution in dimethyl sulfoxide and (ii) 15 M equivalents of IRDye800CW-DBCO (Li-Cor) from a 2 mM stock solution in PBS, concurrently. The reaction mixture was incubated at 25 °C for 16 h at 450 rpm. The site-specifically labeled antibodies were then purified by centrifugal filtration as previously described.

#### Degree of Labeling Determination by UV Spectroscopy

IRDye800CW degree of labeling (DOL_IRDye800CW_) was determined by measuring the absorbance of the antibody conjugates at 280 and 774 nm.
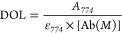
where  and .

CF = correction factor (*A*_280_/*A*_774_), ε_max_ = molar attenuation coefficient at *A*_max_, ε1% = percent molar attenuation coefficient for a 10 mg/mL IgG solution.

### LC ESI-qTOF MS

Immunoconjugate samples in PBS (∼5 μL, 0.7–1.0 mg/mL) were reduced by the addition of the required volume of DTT (100 mM) to yield a 10 mM DTT concentration in each sample. The samples were incubated at 60 °C for 30 min prior to MS analysis. Protein samples were analyzed using a Thermo RSLC coupled to a Bruker maXis. The equivalent of 1–5 μM (1–5 pmol/μL) protein was injected onto the system per run. The loading pump was used at high flow to run linear chromatography gradients. The buffer system comprised Buffer A: 0.1% (v/v) formic acid and Buffer B: 99.9% acetonitrile, 0.1% (v/v) formic acid. Chromatographic separations were achieved using a Fortis C8 column, 100 mm × 2.1 mm, 3 μM particle size, 45 °C at a flow rate of 150 μL/min. Proteins were loaded onto the column and desalted online for 1 min, with eluent diverted to waste. A valve switch directed flow to the mass spectrometer. Proteins were then eluted from the column using a linear gradient from 3% to 70% Buffer B over 12 min. Total run time was 17 min. The eluent was directed into a Bruker maXis 4G, operated in a positive mode. Source conditions had a dry temperature of 200 °C, dry gas 6 L/min, Nebuliser gas 2.5 L/min, capillary 4500 v, and end plate offset 500 v. Data were acquired in an MS scan mode between 800 and 5000 *m*/*z*. Resolution of the instrument was 70,000 at *m*/*z* 922. Data files were assessed in Bruker Data Analysis software. The charge state ruler was used to crudely assess and deconvolute spectra. For more accurate deconvolution, .xy files were exported and analyzed using UniDec software.^31^

### SDS-PAGE

Anti-MT1-MMP immunoconjugates were analyzed using reduced sodium dodecyl sulfate-polyacrylamide gel electrophoresis (SDS-PAGE). Samples were prepared by adding sample buffer (2.5 μL, NuPAGE 4× LDS sample buffer), dithiothreitol (1 μL, NuPAGE 10× Sample Reducing Agent), and deionized water (5.5 μL) to each of the antibody samples (1 μL, 1 mg/mL). The resulting solutions were incubated at 70 °C for 10 min at 450 rpm. Protein samples and molecular weight standards (ThermoScientific PageRuler Unstained Broad Range Protein Ladder) were then loaded on to a 10-well protein gel (4–12% Bis-Tris) and run for 1 h at 200 V in NuPAGE MOPS SDS running buffer. Once complete, the gel was washed three times in water (200 mL, 5 min) before staining using Coomassie Fluor Orange protein stain (50 mL) for 1 h. Destaining was achieved by washing in acetic acid (1 M) for 5 min before finally washing again in water. The gel was scanned using a Typhoon Bioimager for Coomassie Fluor Orange (Cy3, λ_ex_ = 532 nm) and IRDye800 (Cy7, λ_ex_ = 785 nm).

### Zirconium-89 Radiolabeling and Purification

In brief, an aliquot containing approximately 17 MBq of zirconium-89 in oxalic acid (1 M, PerkinElmer) was adjusted to pH 7 by the addition of sodium carbonate (1 M). The resulting solution was added to a solution of DFO-anti-MT1-MMP-IRDye800CW or DFO-IgG-IRDye800CW in PBS (95–101 μL, 2.3–2.4 mg/mL). The reaction mixture was incubated at room temperature for 1 h at 450 rpm, and the radiolabeling efficiency was determined by radio-iTLC using EDTA (50 mM, pH 5.5) as the mobile phase. RICs were purified from the crude reaction mixture by size exclusion chromatography using Sephadex-G50 resin (Sigma-Aldrich), eluting with 100 μL fractions of PBS (pH 7.4) as the eluent. The radiochemical purity of isolated RICs was determined by radio-iTLC as previously described.

### Serum Stability Studies

[^89^Zr]Zr-DFO-anti-MT1-MMP-IRDye800CW (27 μL, 0.1 mg/mL, 0.2 MBq) was added to human serum (500 μL, Sigma-Aldrich, Cat# H4522), mouse serum (500 μL, Sigma-Aldrich, Cat# M5905), or PBS (500 μL, 1×, pH 7.4) and incubated at 450 rpm for 7 days at either 37 °C or 25 °C in triplicate. RIC stability was assessed at 24 h intervals by radio-iTLC as previously described.

### Cell Lines

HT1080 WT (high MT1-MMP, obtained from ATCC) and KO (low MT1-MMP) cell lines were authenticated before use and found to be a 100% match to a HT1080 profile located on the ATCC database (authenticated by NorthGene Limited, Newcastle). Details of creation of the HT1080 MT1-MMP KO cells and validation of the gene and protein knock-down are provided in the Supporting Information Cells were cultured in an RPMI 1640 cell culture medium (Sigma-Aldrich, Cat#: R8758, with l-glutamine and sodium bicarbonate), supplemented with 10% fetal bovine serum (FBS, Gibco), 100 units/mL penicillin, and 10 μg/mL streptomycin (Gibco), in a humidified 5% CO_2_ environment at 37 °C. Cells were harvested and passaged using Trypsin–EDTA solution (Gibco) with a subcultivation ratio of 1:15 every 3–4 days. The cumulative length of the culture was <6 months following retrieval from liquid nitrogen storage. Cells were tested for the absence of mycoplasma at regular intervals.

### *In Vitro* Assays

The immunoreactivity of [^89^Zr]Zr-DFO-anti-MT1-MMP was determined on HT1080 WT cells by linear extrapolation to an infinite antigen excess according to methods described by Lindmo *et al.*(32) Data were background-corrected, and the ratio of the total-to-bound activity was plotted against the inverse of the normalized cell concentration for the linear regression analysis. To determine the binding affinity of [^89^Zr]Zr-DFO-anti-MT1-MMP-IRDye800CW to MT1-MMP, aliquots of 2 × 10^5^ HT1080 WT and HT1080 KO cells were seeded onto 24-well plates in 500 μL of growth medium and allowed to adhere overnight. Cells were then incubated with increasing amounts (2–100 nM) of [^89^Zr]Zr-DFO-anti-MT1-MMP-IRDye800CW for 2 h at 4 °C. The supernatant was removed, and cells were washed twice with PBS (500 μL). Cells were lysed by incubation with 0.1 M NaOH for 30 min ^89^Zr activity in the cell-associated fractions was measured with an automated gamma counter. Binding affinity (dissociation constant [*K*_d_]) was estimated by nonlinear regression analysis with a 1-site total binding model.

### IHC Staining for MT1-MMP

Sections of murine sarcoma tissue were cut from formalin-fixed paraffin-embedded tissue blocks at a thickness of 3 μm and mounted onto Superfrost microscope slides. Sections were deparaffinized in xylene and rehydrated through a graded series of alcohol to deionized water. Controlled antigen retrieval was induced with citrate buffer (pH 6.0) for 40 min at 100 °C. Before RIC incubation, tissue sections were blocked with 4% BSA/PBS (1 mL) for 2 h at 25 °C. A 1:50 dilution of [^89^Zr]Zr-DFO-anti-MT1-MMP-IRDye800CW or [^89^Zr]Zr-DFO-IgG-IRDye800CW in 4% BSA/PBS (1 mL) was added to tissue sections and incubated at 4 °C overnight, followed by washing with PBS (5 × 1 mL, 5 min). Fluorescence and autoradiography images of the resulting sections were obtained using an Amersham Typhoon Bioimager and co-registered to images of adjacent tissue sections that had been stained with 3,3′-diaminobenzidine to visualize MT1-MMP expression and a hematoxylin counterstain.

### *In Vivo* Studies

All mouse studies were carried out in accordance with UK Animals (Scientific Procedures) Act, 1986, under project license P74687DB5 following approval from Newcastle University Animal Ethical Review Body (AWERB). NOD SCID gamma (NSG; NOD.Cg-Prkdcscid Il2rg tm1Wjl/SzJ) mice from an in-house colony (females, *n* = 14, aged 15–18 weeks on the date of RIC injection) were housed in specific pathogen free conditions in individually ventilated cages with sterile bedding, *ad libitum* water, and diet (irradiated no. 3 breeding diet, SDS) in a facility with a 10-h dark cycle and controlled temperature and humidity. Mice were examined and checked daily and weighed weekly to ensure good health. Mice were injected intrafemorally with 20 μL of media containing 5000 of either HT1080 wild-type (WT, MT1-MMP positive, *n* = 7) or HT1080 CRISPR-Cas9 knockout (KO, MT1-MMP negative, *n* = 7) cells. During the procedure, mice were anesthetized by isoflurane inhalation and provided with analgesia (subcutaneous carprofen, 5 mg/kg). Cell lines expressed luciferase (Supporting Information Experimental Methods) to allow monitoring of tumor growth by bioluminescent imaging (BLI) weekly. For imaging, mice were injected interperitoneally with 150 mg/kg D-luciferin (*in vivo* Glo, Promega) and anesthetized with isoflurane prior to measurement of total flux using an IVIS Spectrum (Caliper Life Sciences).

### IVIS Imaging

Two to three weeks after intrafemoral inoculation and confirmation of HT1080 cell tumor growth by BLI, mice were transferred to non-florescent bedding for 1 day and administered either [^89^Zr]Zr-DFO-anti-MT1-MMP-IRDye800CW (*n* = 3 for WT tumors, *n* = 4 for KO tumors) or [^89^Zr]Zr-DFO-IgG-IRDye800CW (*n* = 4 for WT tumors, *n* = 3 for KO tumors) *via* the lateral tail vein with doses of 0.8–1.4 MBq (17–24 μg) in 100 μL of PBS. Mice were imaged under isoflurane anesthesia for fluorescence (excitation and emission filters: 745/800 nm) and Cerenkov luminescence (open filter) using an IVIS Spectrum (PerkinElmer) at 24, 48, and 72 h p.i. After the final imaging session, mice were euthanized by cervical dislocation and organs of interest were removed. The samples were immediately rinsed with water, dried, and then scanned with the IVIS as previously described. IVIS images were analyzed using Living Image version 4.7.2 software (PerkinElmer) by drawing regions of interest (ROIs) in defined areas/organs.

### *Ex Vivo* Biodistribution

After imaging, selected organs, tissues, and blood were transferred into pre-weighed counting tubes. After weighing the filled counting tubes, the activity in each sample was measured with a gamma counter. Counts per minute were converted into activity units (MBq) using a calibration curve generated from known standards. These values were decay-corrected to the time of injection and normalized to the injected activity. The percentage of the injected activity per gram (%IA/g) of each sample was calculated. Tissues of interest were then flash-frozen with dry ice and stored at −80 °C until required for further processing.

### Statistical Analysis

All statistical and regression analyses were performed using GraphPad Prism v9 (GraphPad Software, San Diego, CA, USA). A confidence interval of 95% (*P* < 0.05) was considered statistically significant. One-way ANOVA followed by Tukey’s post hoc test was used to compare multiple groups. All data were obtained in at least triplicate, and results were reported and graphed as mean ± standard deviation, unless stated otherwise.

## Supporting Information Available

Additional experimental methods and data from mass spectrometry, radiolabeling, and *in vitro* experiments (PDF)

## Abbreviations

Ab: antibody
DBCO: dibenzocyclooctyne
DFO: desferrioxamine
DOL: degree of labeling
IgG: immunoglobulin G
iTLC: instant thin layer chromatography
IV: intravenous
KO: knock out
LCMS: liquid chromatography mass spectrometry
NIR: near infrared
NOD: non-obese diabetic
NSG: NOD SCID gamma
PBS: phosphate buffered saline
PET: positron emission tomography
RCP: radiochemical purity
RCY: radiochemical yield
RIC: radioimmunoconjugate
rpm: revolutions per minute
SCID: severe combined immunodeficient
SDS-PAGE: sodium dodecyl sulfate-polyacrylamide gel electrophoresis
WT: wild type
^89^Zr: zirconium-89
