# Supplementary material for: Synthesis and In Vivo Evaluation of a Site-specifically Labeled Radioimmunoconjugate for Dual-Modal (PET/NIRF) Imaging of MT1-MMP in Sarcomas
Source: Bioconjug Chem. 2022 Jul 22;33(8):1564–73. doi: 10.1021/acs.bioconjchem.2c00306 (PMC9389524; doi:10.1021/acs.bioconjchem.2c00306)
Supplement: Supplementary file 1 — bc2c00306_si_001.pdf [file bc2c00306_si_001.pdf]

Supporting Information

Synthesis and *In Vivo* Evaluation of a Site-Specifically Labelled Radioimmunoconjugate for Dual-Modal (PET/NIRF) Imaging of MT1-MMP in Sarcomas

Toni A. Pringle[1], Corey D. Chan[3,4], Saimir Luli[5], Helen J. Blair[4,6], Kenneth S. Rankin[3,4,*], James C. Knight[1,2,*]

[1] School of Natural and Environmental Sciences, Newcastle University, Newcastle Upon Tyne, NE1 8QB.

[2] Newcastle Centre for Cancer, Newcastle University, Newcastle upon Tyne, NE1 7RU.

[3] North of England Bone and Soft Tissue Tumour Service, Newcastle upon Tyne Hospitals NHS Foundation Trust, Freeman Road, Newcastle upon Tyne, NE7 7DN.

[4] Translational and Clinical Research Institute, Newcastle University, Newcastle upon Tyne, NE1 7RU.

[5] Preclinical In Vivo Imaging, Translational and Clinical Research Institute, Newcastle University, Newcastle upon Tyne, NE2 4HH.

[6] Wolfson Childhood Cancer Research Centre, Newcastle upon Tyne, NE1 7RY.

[*] **To whom correspondence should be addressed:**

Dr. James C. Knight
Bedson Building
School of Natural and Environmental Sciences
Newcastle University
Newcastle Upon Tyne
NE1 7RU
Tel: +44 (0)191 2088542
Email: james.knight2@newcastle.ac.uk

Contents

SUPPLEMENTARY FIGURES 3

SUPPLEMENTARY EXPERIMENTAL METHODS 14

Cryostat Sectioning 14

Flow Cytometry 14

Protein Extraction and Western Blot 15

MT1-MMP Knock Out 17

Fluorescence Activated Cell Sorting and Single Cell Seeding 18

Primer Design 18

PCR and Gel Electrophoresis 19

Sanger Sequencing 20

Quantitative Reverse Transcription PCR 21

Transduction of HT1080 WT and KO cells with pSLIEW virus particles 22

SUPPLEMENTARY FIGURES

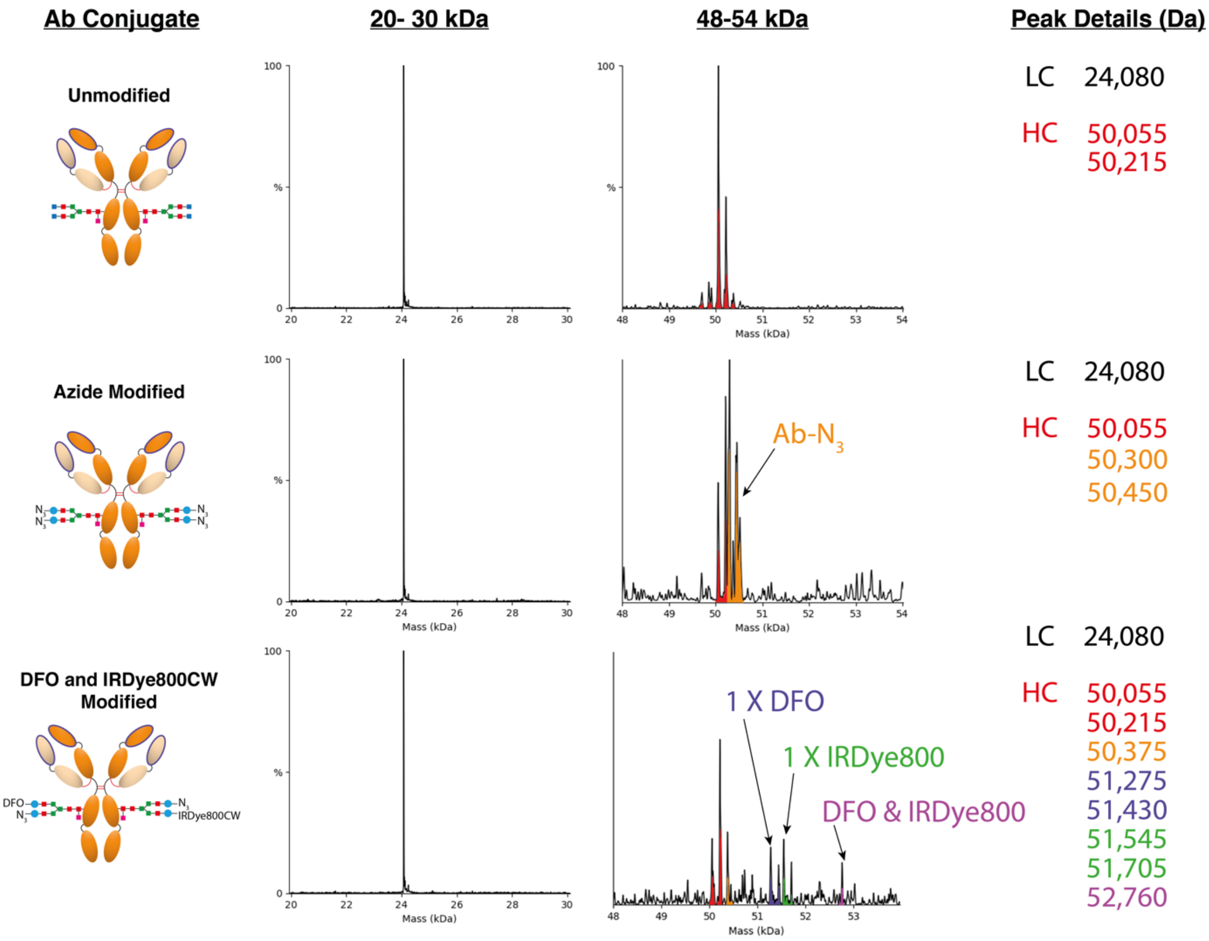

Figure S1. LC ESI-qTOF MS analysis of anti-MT1-MMP dual-modal conjugates. Spectra at 20-30 kDa shows the light chain (LC), the mass of which remains constant throughout the conjugation at 24,080 Da. The spectra at 48-54 kDa shows the heavy chain of the azide modified (orange) and DFO/IRDye800CW modified Ab (blue/green/purple) increasing in mass compared to the unmodified Ab (red).

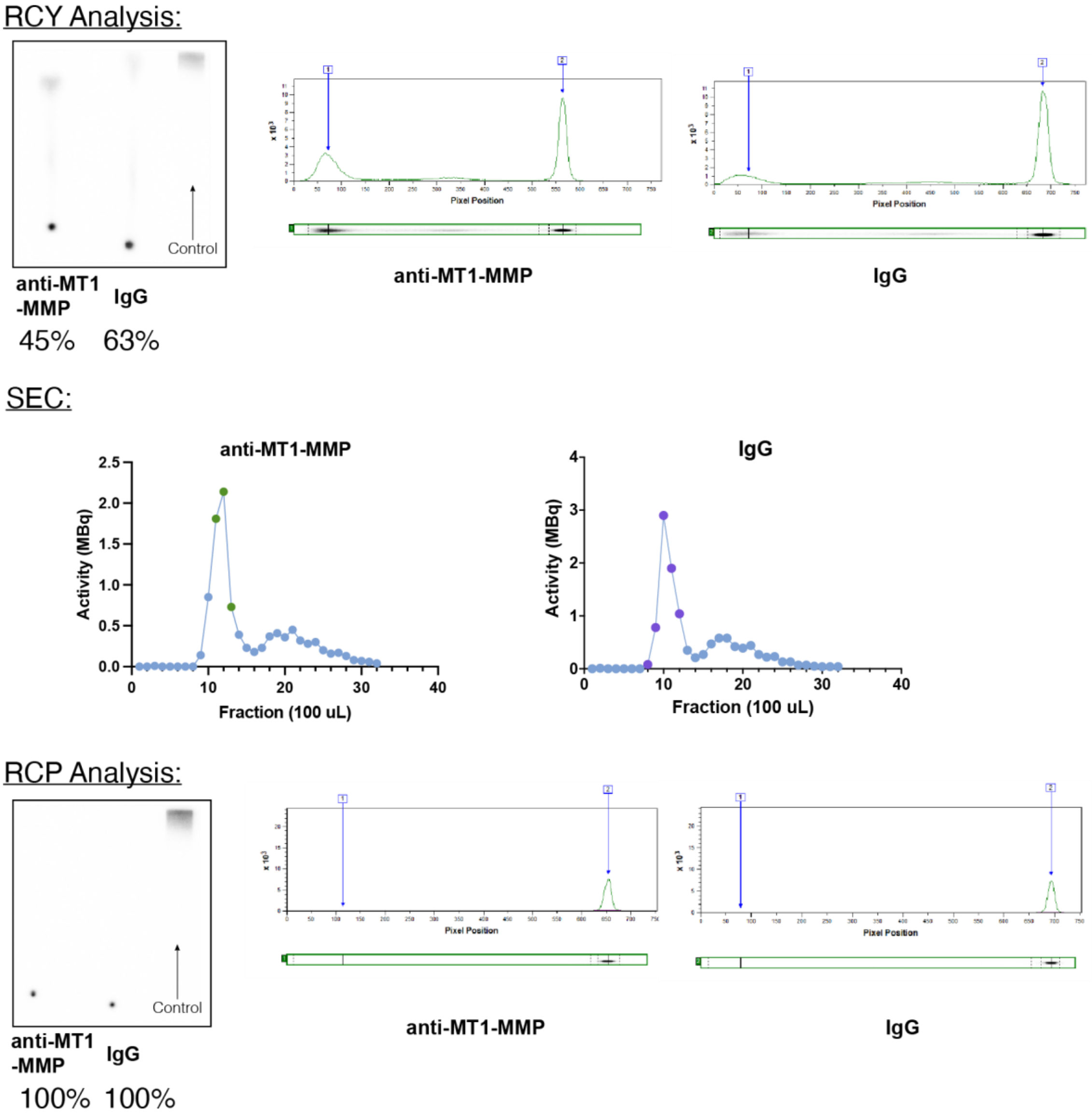

Figure S2. Radiochemical analysis of [^{89}Zr]Zr-DFO-anti-MT1-MMP-IRDye800CW and [^{89}Zr]Zr-DFO-IgG-IRDye800CW conjugates. Top) Radiochemical yield (RCY) radio-iTLC and corresponding profile. Middle) Size exclusion chromatography (SEC) profiles with relevant fractions highlighted in green/purple. Bottom) Radiochemical purity (RCP) radio-iTLC and corresponding profile following SEC.

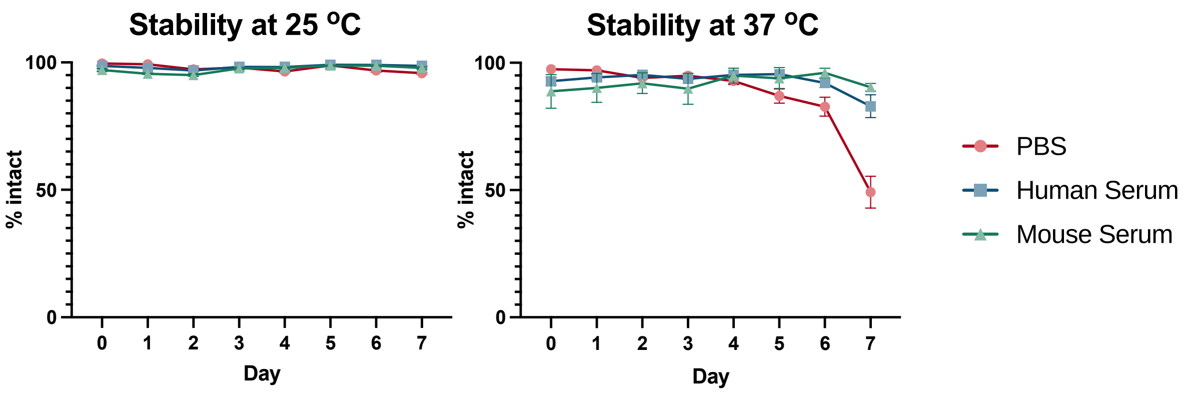

Figure S3. Stability of the [^{89}Zr]Zr-DFO-anti-MT1-MMP-IRDye800CW conjugate in PBS (blue), human serum (purple) and mouse serum (pink) over 7 days at either 25 or 37 °C.

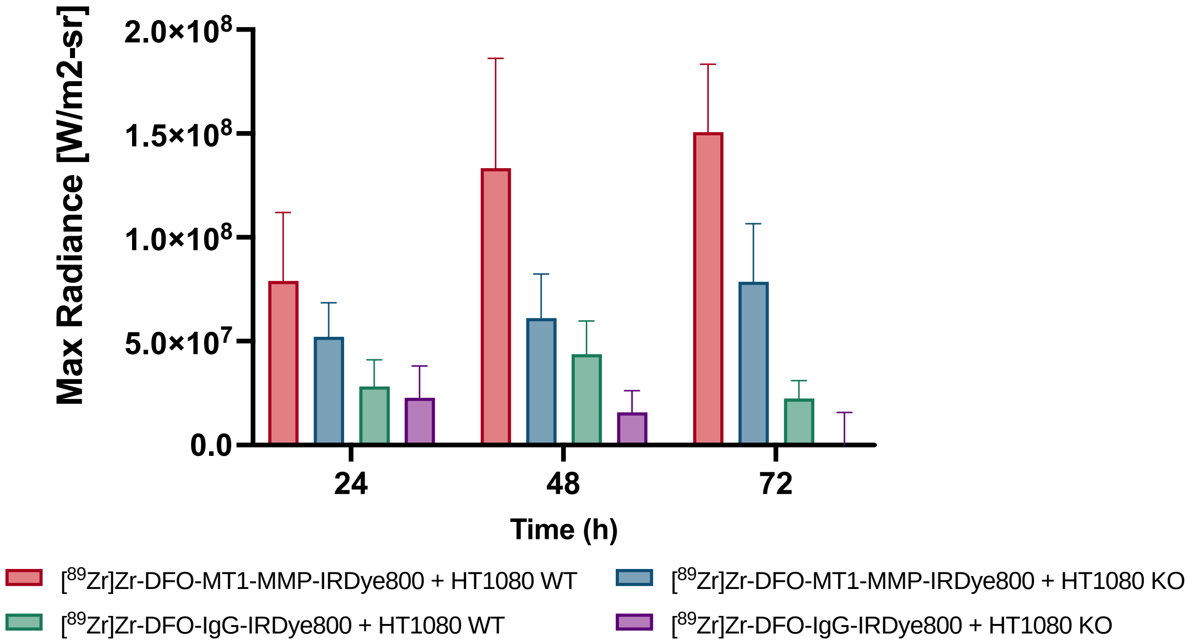

Figure S4. ROI analysis of fluorescence images showing tumour uptake across all groups at 24, 48 and 72 h p.i. of either [^{89}Zr]Zr-DFO-anti-MT1-MMP-IRDye800CW or [^{89}Zr]Zr-DFO-IgG-IRDye800CW in mice bearing either HT1080 WT or KO tumours. Error bars represent SEM.

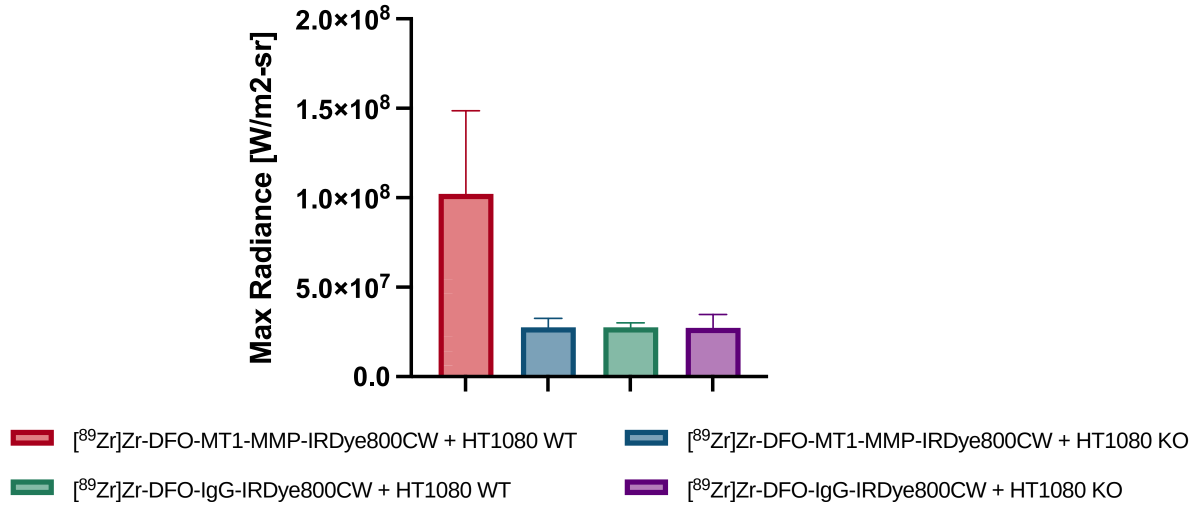

Figure S5. ROI analysis of *ex vivo* fluorescence images showing inoculated femur uptake across all groups at 72 h p.i. of either [^{89}Zr]Zr-DFO-anti-MT1-MMP-IRDye800CW or [^{89}Zr]Zr-DFO-IgG-IRDye800CW in mice bearing either HT1080 WT or KO tumours. Error bars represent SEM.

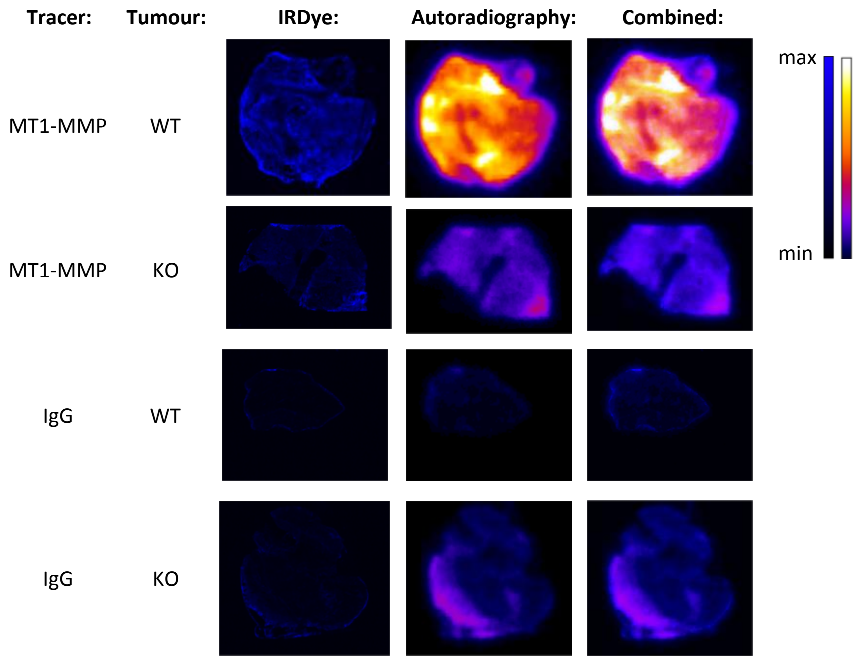

Figure S6. Representative fluorescence (IRDye800) and autoradiography images of sections of invaded muscle around the inoculated femur of HT1080 WT or KO tumour bearing mice injected with [^{89}Zr]Zr-DFO-anti-MT1-MMP-IRDye800CW or [^{89}Zr]Zr-DFO-IgG-IRDye800CW.

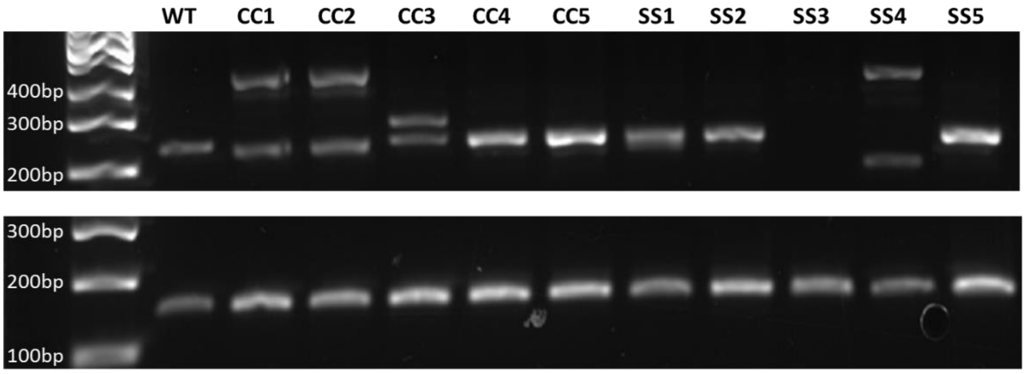

Figure S7. Gel electrophoresis images for the HT1080 KO clones alongside a 100bp DNA ladder to determine the size of the products. Primers for the 18s rRNA were used as a housekeeper with a size of 180 bp. A range of genomic mutations within the MMP14 gene were observed, with larger amplicons indicative of possible insertions and smaller amplicons suggestive of deletions. The PCR products were separated out by electrophoresis in 1.5% agarose gel, containing GelRed DNA binding dye detect the bands on UV imaging.

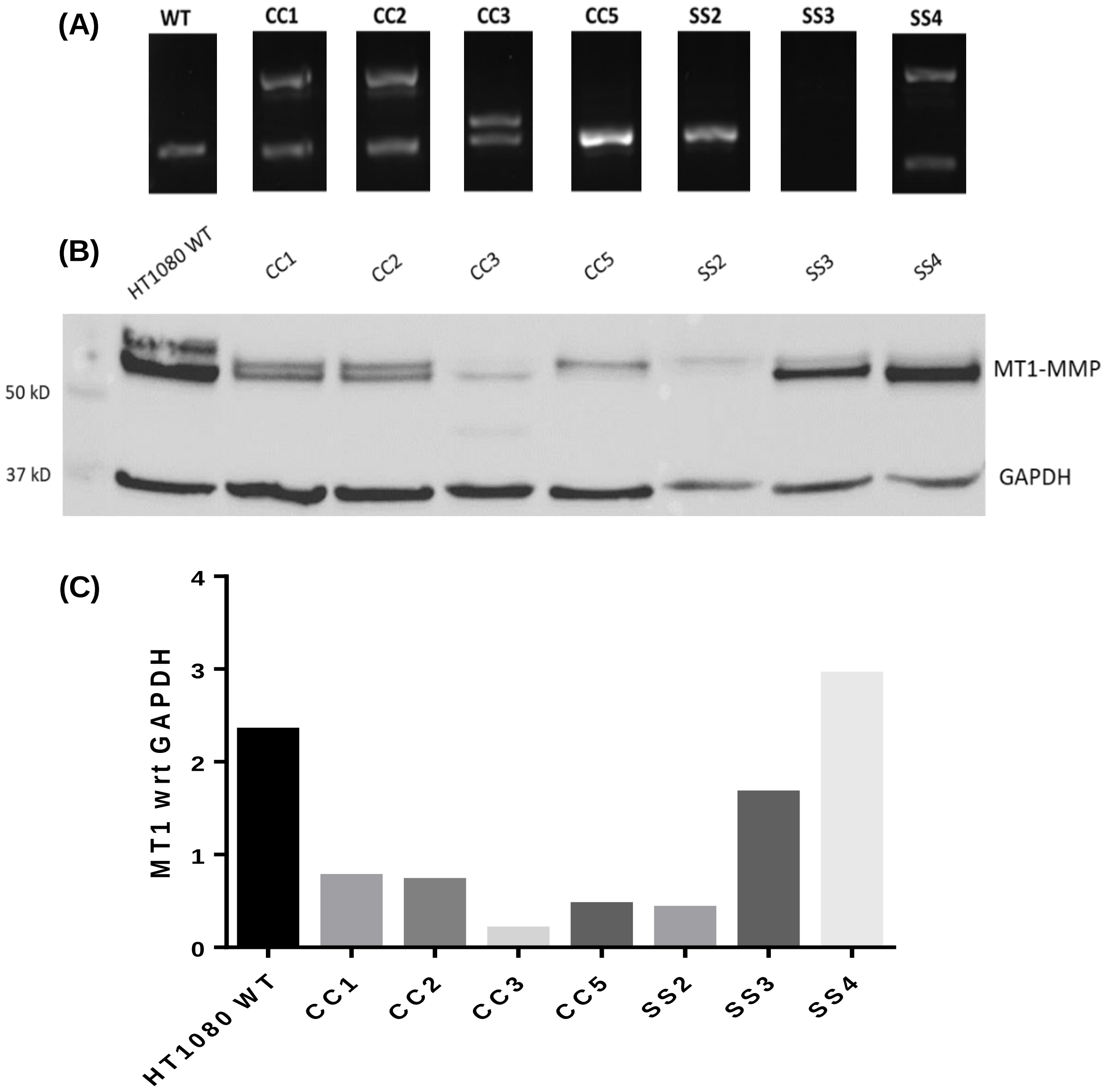

Figure S8. Protein expression across seven HT1080 KO clones on western blot. KO clones had total protein extracted and were analysed by western blotting with primary antibodies against MT1-MMP and GAPDH (B); the different MT1-MMP bands indicate the inactive and active form of the peptide, prior to and following cleavage of the pro-peptide domain by furin before insertion into the plasma membrane. The corresponding gel electrophoresis data is presented above the blot for reference (A). The relative protein expression wrt GAPDH was quantified via Image J (C). HT1080 KO clone CC3 was taken forwards as HT1080 KO cell line. Faint MT1-MMP protein expression can still be seen in the CC3 clone on western blot, likely representing a non-functional protein following CRISPR induced genetic mutation, given the lack of cell surface expression on flow cytometry.

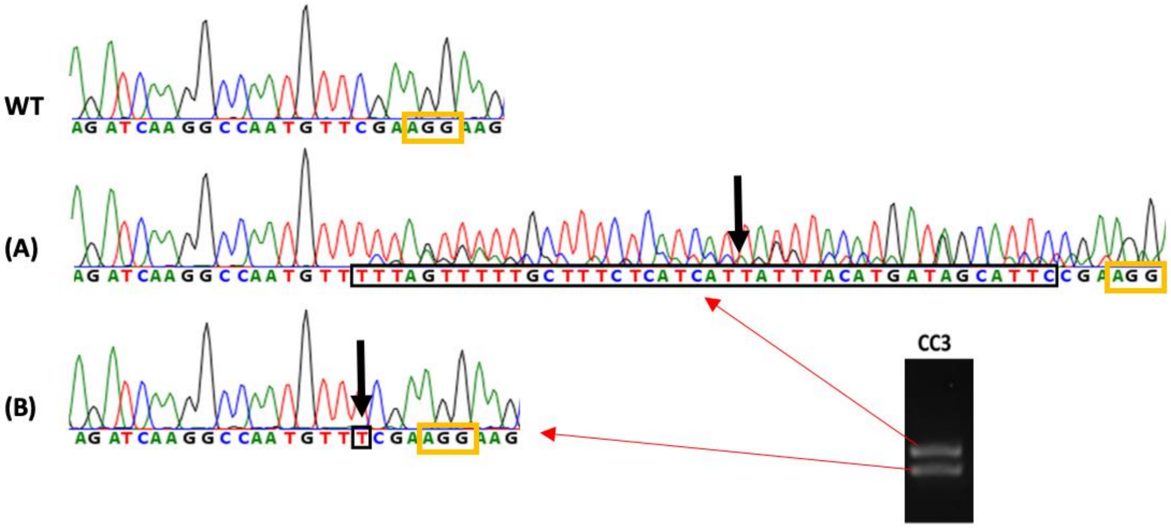

Figure S9. Sanger sequencing showed genetic alterations within the MMP14 gene of HT1080 KO CC3 clone. The two different mutations were identified labelled (A) and (B) aligned to the WT. Black vertical arrows point to the mutations which are outlined in black boxes. The red arrows show which mutation corresponds to each PCR band in the PCR electrophoresis. The PAM sequence (AGG) within the target site is outlined in yellow for reference. The sequencing data was analysed and aligned using DNADynamo software, and showed two different allelic mutations, with a 42 bp insertion (A) and a 1 bp insertion (B), both at base position 10,444 within the MMP14 gene.

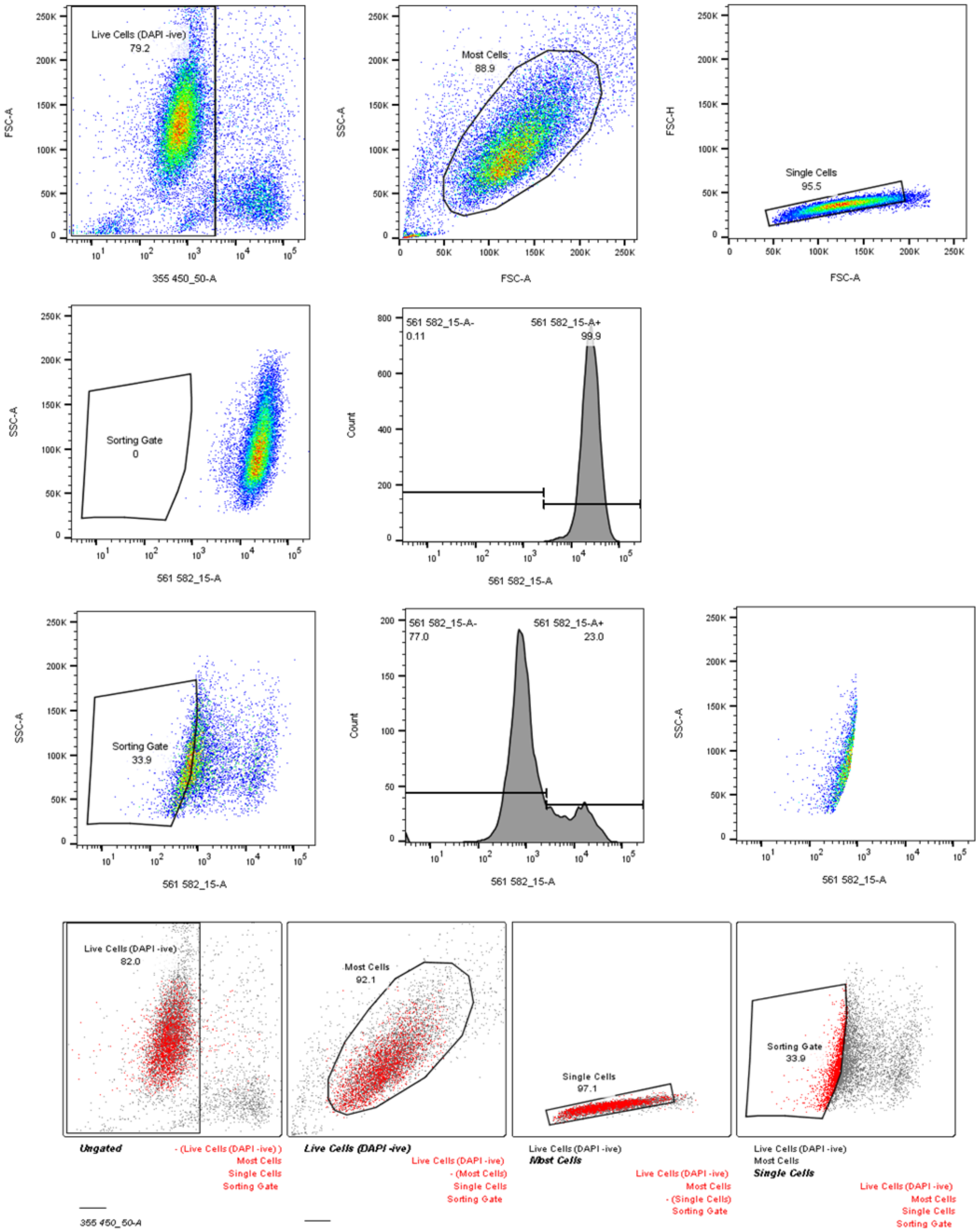

Figure 10. Fluorescence activated cell sorting of HT1080 KO population. HT1080 KO cells were sorted and seeded as single cells into a 96 well plate, using above parameters on BD FACSAria Fusion Sorter. Live cells were gated based on absence of DAPI stain, doublet discrimination via FSC-H, and then gated based on absence of

MT1-MMP cell surface expression compared to WT control. Selected cells are displayed in red.

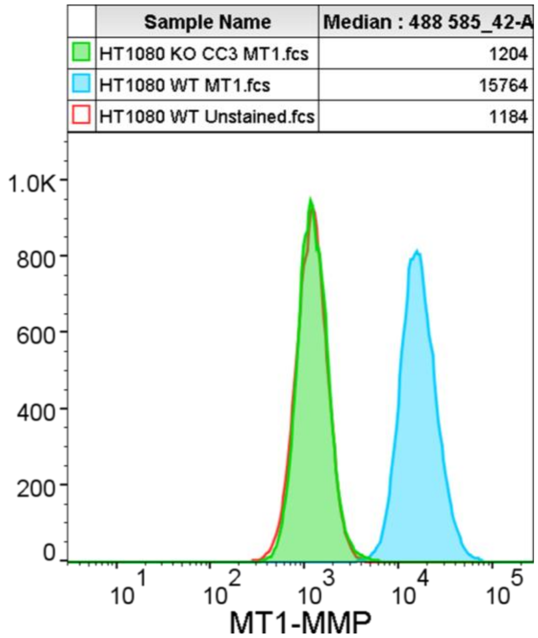

Figure S11. Flow cytometry demonstrated negative MT1-MMP surface expression in HT1080 KO cell line (clone CC3). Cells were stained with MT1-MMP-PE Ab conjugate and analysed on FACS Canto II, using 488 585/42 laser. HT1080 WT showed strong MT1-MMP signal (MFI = 15764) compared to the HT1080 KO and unstained samples (MFI = 1204 and 1184 respectively).

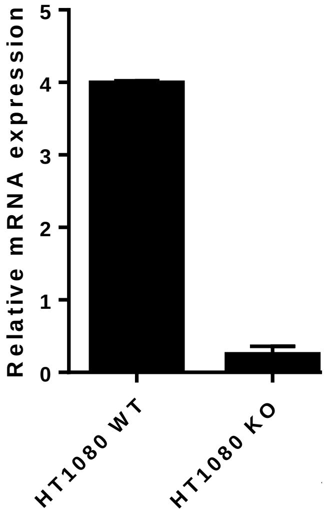

Figure S12. Relative MT1-MMP mRNA levels in the WT and KO populations. qRT-PCR analysis was performed on the QuantStudio 7 Flex Real-Time PCR System, using a 10 µL reaction volume using the standard setting for 40 cycles. The relative mRNA expression across HT1080 WT and HT1080 KO was calculated with respect to HPRT1 using the ΔCt method. The mRNA fold expression change between WT and KO populations was then calculated using the comparative threshold cycle (Ct) $2^{-(\Delta\Delta Ct)}$ method, normalised to the expression of the HPRT1 gene. Mean ± 95% CI's.

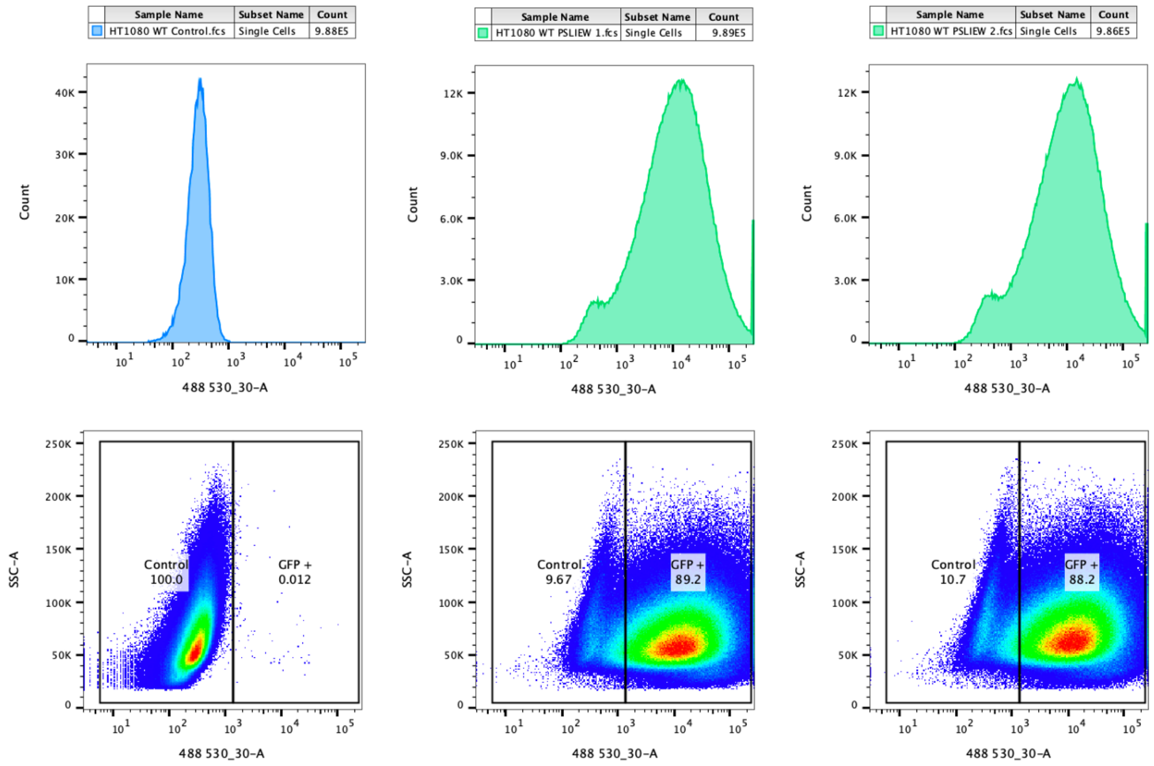

Figure S13. Flow cytometry results showing 89.2% efficiency from the transduction of HT1080 WT cells with pSLIEW virus.

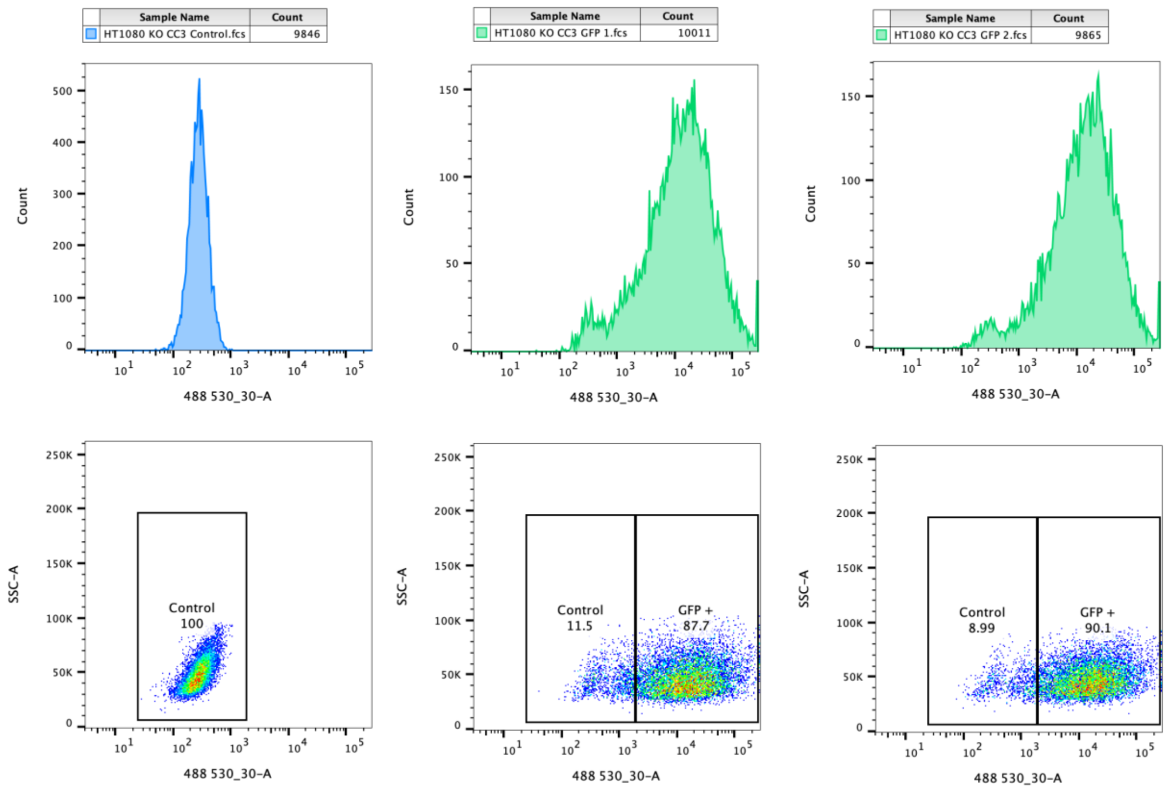

Figure S14: Flow cytometry results showing 90.1% efficiency from the transduction of HT1080 KO cells with pSLIEW virus.

SUPPLEMENTARY EXPERIMENTAL METHODS

Cryostat Sectioning

Following gamma counting analysis, the invaded muscle surrounding the inoculated femur was frozen in dry ice before being embedded in OCT and sectioned at -20 °C on a Leica cryostat at 5 μm thickness. The sections were attached to a microscope slide which was stored at -80 °C until fluorescence imaging and autoradiography.

Flow Cytometry

Cells in a T75 flask were washed with PBS (10 mL) then of flow buffer (500 mL PBS, EDTA (2.5 mL 0.2 mmol/L) and MACS BSA Stock Solution (2.5 mL)) was added, incubated at 37 °C for 10 minutes, lifted and then counted. Cell suspensions containing $1{\times}10^6$ cells in flow buffer (100 μL) were transferred to FACS tubes. Seroblock (5 μL,

Bio-rad) was added per tube and incubated on ice for 15 minutes. Human MMP-14 PE-pre-conjugated antibody (20 µL, R&D systems) was added and cells incubated in the dark and on ice for 50 minutes. Two wash cycles were performed then the final pellet was reconstituted in flow buffer (500 µL) and analysed using the BD FACS Canto II Analytical Fluorescence Flow Cytometer, with the 488nm (585/42) excitation laser. Samples were analysed at a low flow rate for 100,000 total events. Flow cytometry data was analysed using FlowJo V10 Software, doublets were discriminated using FSC-H, and a histogram with log scale generated against 488: 585/42-A. The median fluorescence intensity (MFI) for each cell line was calculated by (MFI sample – MFI unstained control). MFI values were obtained using the statistical function within FlowJo software.

Protein Extraction and Western Blot

Protein Extraction and Quantification

Cells were washed with PBS and 500µL RIPA solution containing protease inhibitors was added per T-75 flask and incubated on ice for 10 minutes. After collection with a cell scraper (Corning Costar 3010), lysate was ultrasonicated for 10 seconds at amplitude 10 and centrifuged at 14,000 rpm for 10 minutes at 4 °C. The supernatant was retained.

Quantification was performed using the Pierce BCA Protein Assay Kit as per the manufacturer's protocol. Samples were diluted 1:5 with PBS. 10 µL of each BSA standard and protein sample were pipetted into a 96 well plate in duplicate. BCA working reagent (200 µL) was prepared and added to each well. The plate was covered and incubated at 37 °C for 30 minutes, then the absorbance measured at 570 nm using a BioRad Model 680 Plate Reader. Concentrations of the unknown samples were calculated in Microsoft Excel using a linear trend-line equation derived from the BSA standards.

Table S1: Antibodies used for western blotting.

Antibody	Concentration	Solution	Manufacturer and Ab Details
Primary MT1-MMP Ab	1:1000	TBS-T	EMD Millipore<br>Mouse Anti-MMP14 monoclonal (MAB3328)
Anti-mouse secondary Ab	1:2000	5% Milk/TBS-T	Dako<br>Polyclonal Goat Anti-Mouse Immunoglobulin HRP (P0447)
Primary GAPDH Ab	1:10000	TBS-T	Cell Signalling<br>Rabbit GAPDH monoclonal (D16H11)
Anti-Rabbit Secondary Ab	1:2000	5% Milk/TBS-T	Dako<br>Polyclonal Goat Anti-Rabbit Immunoglobulin HRP (P0448)

Table S2: Reagents used for protein extraction and western blotting.

Reagent	Contents/Details
RIPA solution	• 490 µL Pierce RIPA Buffer (Thermo Scientific)<br>• 5 µL Halt Protease Inhibitor Single-Use Cocktail 100X (Thermo Scientific)<br>• 5 µL 0.5 M EDTA Solution 100X (Thermo Scientific)
Pierce BCA Protein Assay Kit (Thermo Scientific)	• BCA Reagent A, 500 mL<br>• BCA Reagent B, 25 mL<br>• Albumin Standard Ampules, 2 mg/mL, 10 x 1 mL
Protein Ladder	• Precision Plus Protein Kaleidoscope (Bio-Rad)
1 x Running Buffer	• 25 mL 20x BOLT MES SDS Running Buffer (Novex)<br>• 475 mL of distilled water
1 x Transfer Buffer	• 120 mL of 10x Tris/Glycine Buffer (Bio-Rad)<br>• 240 mL methanol<br>• 840 mL distilled water
1 x TBS-T	• 100 mL 10x Tris buffered saline (Bio-Rad)<br>• 900 mL distilled water<br>• 1 mL Tween 20 (Sigma-Aldrich)
5% Milk/TBS-T Solution	• 2.5 g dried skimmed milk powder (Marvel)<br>• 50 mL TBS-T
Sample Buffer	• BOLT LDS Sample Buffer 4x (Novex)

Reducing Agent	• BOLT Sample Reducing Agent 10x (Novex)
Pre-cast gel	• 4-12% Bis-Tris pre-cast gel (Bio-Rad Criterion XT)
Membrane	• Nitrocellulose Membrane 0.45 µm (Bio-Rad)
ECL Substrate	• Clarity Western ECL Substrate chemoluminescence stain (Bio-Rad)

Protein Electrophoresis and Membrane Probing

Quantified protein samples were prepared at a concentration of 40 µg in 26 µL of RIPA buffer. Sample buffer (10 µL) and reducing agent (4 µL) was added to each sample, heated at 100 °C for 10 minutes and centrifuged at 14,000 rpm for 30 seconds at RT. Samples were loaded into wells of a pre-cast gel alongside a protein ladder (3 µL), in 1 X running buffer (500 mL) and ran at 100V for 120 minutes. Proteins in the gel were transferred onto a membrane by placing into a transfer sandwich and ran at 100 V for 55 minutes in pre-chilled transfer buffer. The membrane was blocked in 5 % milk on a rocker at RT for 1 hour.

The membrane was washed and anti-MT1-MMP primary antibody solution was added to the membrane, covered and incubated on a rocker at 4 °C overnight. The membrane was washed and an anti-mouse secondary antibody was added and incubated at RT for 1 hour. The membrane was washed again before adding ECL substrate (1 mL) and incubating for 3 minutes. The membrane was imaged using Bio-Rad ChemiDoc Imaging System before washing and repeating the process for primary GAPDH antibody with anti-rabbit secondary antibody.

MT1-MMP Knock Out

MT1-MMP KO in the HT1080 cell line was achieved using the Liopfectamine MessengerMax protocol and reagents (Invitrogen). The day before transfection, $1{\times}10^5$ cells were seeded in a 24 well plate in triplicate. Two wells were transfected, and one remained as an untreated control. On the day of transfection, appropriate cell confluency (60-70%) was observed, old media was aspirated and replaced with fresh media (500 µL). The following solutions were prepared: Opti-MEM 1 x medium (100 µL, Life Technologies) and GeneArt™ Platinum™ Cas9 Nuclease (2 µL, 1 µg/µL, Invitrogen) was added to sgRNA (1 µL, 3 nmol, MMP-14 TrueGuide Syn sgRNA (Invitrogen CRISPR906767) suspended in Tris-EDTA buffer solution (30 µL, Sigma-

Aldrich). Liopfectamine MessengerMax reagent (8.25 µL, Invitrogen) and Opti-MEM 1 x medium (137.5 µL, Life Technologies) was then added and the contents gently mixed by vortexing, followed by incubation at RT for 15 minutes to allow CRISPR/lipid complexes to form. 60 µL of the preparation was then added to each treatment well. The plate was gently agitated and incubated at 37 °C, 5 % CO_2 for 40-48 hours. Cell populations were grown to confluency.

Fluorescence Activated Cell Sorting and Single Cell Seeding

Cells were prepared for FACS sorting as per the flow cytometry protocol, with $4.5x10^6$ cells per flow tube stained with Human MMP-14 PE-pre-conjugated (R&D systems) antibody. DAPI (5 µL, 1:100, Sigma-Aldrich) was added to each tube 5 minutes prior to sorting. Sorting was performed on the BD FACSAria Fusion Sorter (100 µm nozzle) at 20 psi using the highest purity setting (0-32-0) with the 561 nm PE excitation laser (582/15). The appropriate sorting gates were set to set to discard dead cells and doublets. MT1-MMP negative cells were then seeded as single cells into wells of a 96 well plate, with each well containing 200 µm of conditioned media (prepared from 1-part recovered media to 1-part fresh media). Each well was immediately assessed, and any well with no cell, or greater than one cell was marked for discard. The plate was incubated at 37 °C for 2 weeks, and media was replaced every 3 days. When the number of single cell populations had reached 60-80% confluency in the 96 well plate, they were transferred to a 48 well plate and continuously cultured.

Primer Design

<u>Genomic DNA Primers</u>

The nucleotide sequence of the MMP14 gene (NG_046989.1) was obtained from the National Centre for Biotechnology (NCBI) Human Genome Resources in FASTA format. The target sequence of the TrueGuide Synthetic sgRNA CRISPR906767 (AGATCAAGGCCAATGTTCGA) was identified within the MMP-14 reference sequence. A nucleotide sequence 200 bp upstream and 200 bp downstream of the target site was isolated and inputted into Primer 3 and Primer-Blast online tools. An amplicon length between 200-250 bp was specified. Generated primer pairs were

analysed for suitability based on basic design rules. Suitable pairs were selected, ensuring the target site was centrally located within the amplicon.

	Oligonucleotide Sequence (5'->3')	Length	Start Base	Stop Base	Tm (°C)	GC%	Self-compleme-ntarity
**Forward Primer**	CACTGATCCCA ATCCTCGCA	20	118	137	59.82	55.00	4.00
**Reverse Primer**	CCCTGCATAAG CACAATGGG	20	346	327	59.25	55.00	6.00

Product Size: 229 bps

qRT-PCR Primers

The MT1-MMP mRNA transcript reference sequence was identified using Ensembl (NM_004995.4) and inputted into Primer-Blast (NCBI). Primers for qRT-PCR were designed away from the CRISPR sgRNA target site to prevent any mutations from preventing primer binding. An amplicon length between 80-150 bp was specified. Primers were designed to include an exon-exon span to reduce amplification of gDNA contamination.

	Oligonucleotide Sequence (5'->3')	Length	Start Base	Stop Base	Tm (°C)	GC%	Self-complementarity
**Forward Primer**	CCGGCCTTCT GTTCCTGATA	20	1136	1155	59.17	55.00	4.00
**Reverse Primer**	CAGCGCTCCTT GAAGACAAAC	21	1252	1232	60.07	52.38	6.00

Product Size: 117 bps

PCR and Gel Electrophoresis

Genomic DNA Extraction of KO Clones

Cell pellets were prepared from confluent single cell colonies in a 12 well plate. Genomic DNA extraction was performed using the Invitrogen PureLink Genomic DNA

Mini Kit exactly as stated in the manufacturer's protocol (K1820-01) for Mammalian Cell Lysate.

PCR:

Genomic DNA for each clone was prepared at a concentration of 20 ng/µL by dilution in UltraPure DNase/RNase-Free water. Forward and Reverse Primer mixes were prepared at a working concentration of 10 µM. Primers against the 18s rRNA gene (Sigma) were used as a housekeeper (Forward: 5'-GTAACCCGTTGAACCCCATT, Reverse: 5' – CCATCCAATCGGTAGTAGCG). Each reaction mix consisted of sample gDNA (1 µL) and the master-mix (24 µL, Platinum II Hot-Start Green PCR Master Mix 2X (Invitrogen), Forward/Reverse Primers and DNase/RNase-Free water). PCR was performed in the Techne Prime Thermocycler using the following programme: Lid preheated to 105 °C, initial denaturation 94 °C for 2 minutes, followed by 30 cycles of: 94 °C (30s), 58 °C (30s), 68 °C (30s) with a 4 °C final hold phase.

Agarose Gel Preparation and Electrophoresis

1.5 % agarose gel was prepared using agarose powder (1.5 g, Sigma-Aldrich) in 0.5 x tris-borate-EDTA (100 mL, TBE) buffer diluted in distilled water from 10 x stock. The agarose solution was combined with PAGE GelRed (10 µL, 10,000 X) nucleic acid binding dye and cast into a gel tray with two 16 tooth combs. Gel was set at 4 °C for 1 hour, then placed in a horizontal gel tank in 0.5 x TBE buffer. PCR (15 µL) product was pipetted into the wells alongside 100 bp DNA ladder (15 µL, Invitrogen) and electrophoresed for 60 minutes at 100 V. Imaging performed using the Bio-Rad ChemiDoc Imaging System.

Sanger Sequencing

Band Excision and Gel Purification

In a dark room, bands were excised with a sterile scalpel over a UV source and immediately purified and prepared for sequencing using the QIAquick Gel Extraction

Kit as per the protocol. DNA was eluted with Buffer EB (30 µL). The purified gel products and forward primer (100 µL, 10 µM) were labelled and sequenced by Eurofins GATC service. The results were analysed in DNADynamo software.

Quantitative Reverse Transcription PCR

RNA Extraction

RNA Extraction was performed using the Qiagen RNeasy Mini Kit (74104) as per the manufacturer's protocol for animal cells. Pellets containing 3.5×10^{6} cells were prepared and Buffer RLT (350 µL) was added. Lysate homogenisation was performed using QIAshredder spin columns (Qiagen). The optional step to remove excess Buffer RPE was performed. RNA was eluted using RNase-free water (30 µL) and quantified on using the NanoDrop™ 1000 Spectrophotometer (ThermoScientific).

cDNA Synthesis

For each sample, 1000 ng of RNA was prepared in RNase-free water (12.7 µL). A master-mix was prepared (M-MLV RT Reaction Buffer 5 (Promega), dNTP Mix (10 µM, Thermo Scientific), Oligo d(T)16 (50 µM Invitrogen) diluted 1:5 in RNase-free water, M-MLV Reverse Transcriptase (Promega)) and briefly vortexed. RNA samples were placed at 65 °C for 2 minutes then the master-mix (7.3 µL) was added to each sample. Samples were placed at 37 °C for 1 hour and then increased to 100 °C for 10 minutes.

Derivation of Primer Efficiency

Both primer pairs were validated by running a 5 X serial dilution with cDNA from HT1080 WT cells. Standard curves were generated for both sets of primers and the efficiencies were within the range (90-110%) with r^2 value >0.980.

qRT-PCR

qRT-PCR was performed using the QuantStudio 7 Flex Real-Time PCR System (ThermoScientific) using a standard instrument run for 40 cycles. MT1-MMP and HPRT1 master-mixes were prepared using Platinum SYBR Green qPCR SuperMix protocol. 8 µL of each master-mix and 2 µL of undiluted cDNA were added in quadruplet to wells of a 384 well PCR plate. The plated was sealed with optical adhesive and briefly centrifuged. HPRT1 primers (Sigma) had the following sequences: Forward: 5'–AGAATGTCTTGATTGTGGAAGA, Reverse: 5'–ACCTTGACCATCTTTGGATTA. Data was processed using the QuantStudio Real-Time PCR Software v1.3 and analysed using the comparative threshold cycle (Ct) 2 –(ΔΔCt) method, normalised to the expression of the HPRT1 gene.

Transduction of HT1080 WT and KO cells with pSLIEW lentivirus

HT1080 WT and KO cells (100,000 cells per well) were seeded into a 6-well plate with growth medium (2 mL) and incubated at 37 °C overnight. pSLIEW virus (20 µL) was added to each well and mixed thoroughly. Viral transduction was performed by spinfection at 900 × g for 50 minutes at 32 °C before incubating overnight at 37 °C overnight. The media containing the virus was aspirated off and the cells were washed with PBS before adding fresh media (2 mL). Once cells had reached confluency, they were transferred into increasingly larger flasks until an appropriate cell number was achieved to perform flow cytometry.
